# A Microbiota-Directed Food Intervention for Undernourished Children

**DOI:** 10.1056/NEJMoa2023294

**Published:** 2021-04-07

**Authors:** Robert Y. Chen, Ishita Mostafa, Matthew C. Hibberd, Subhasish Das, Mustafa Mahfuz, Nurun N. Naila, Md.Munirul Islam, Sayeeda Huq, Md.Ashraful Alam, Mahabub Uz Zaman, Arjun S. Raman, Daniel Webber, Cyrus Zhou, Vinaik Sundaresan, Kazi Ahsan, Martin F. Meier, Michael J. Barratt, Tahmeed Ahmed, Jeffrey I. Gordon

**Affiliations:** 1 Edison Family Center for Genome Sciences and Systems Biology, Washington University School of Medicine, St. Louis, MO 63110 USA; 2 Center for Gut Microbiome and Nutrition Research, Washington University School of Medicine, St. Louis, MO 63110 USA; 3 International Centre for Diarrhoeal Disease Research, Bangladesh (icddr,b), Dhaka 1212, Bangladesh; 4 Department of Pathology and Immunology, Washington University in St. Louis School of Medicine, St Louis, MO 63110 USA

## Abstract

**BACKGROUND:**

More than 30 million children worldwide suffer from moderate acute malnutrition (MAM). Current treatments have limited effectiveness and much remains unknown about pathogenesis. Children with MAM exhibit perturbed development of their gut microbiota.

**METHODS:**

Slum-dwelling Bangladeshi children, aged 12 to 18 months, with moderate acute malnutrition (n=124) received a microbiota-directed complementary food (MDCF-2) or an existing ready-to-use supplementary food (RUSF), twice daily for three months followed by a 1-month period of monitoring. We obtained weight-for-length, weight-for-age, and length-for-age Z-scores and mid-upper arm circumference at baseline and fortnightly, through four months. We compared the rate of change of these related phenotypes between baseline and three months, and between baseline and four months. We also measured levels of 4,977 proteins in plasma plus 209 bacterial taxa in fecal samples.

**RESULTS:**

118 children completed the intervention (n=59/arm). The rate of change in weight-for-length Z-score (β-WLZ), weight-for-age Z-score, and mid upper arm circumference is consistent with a benefit of MDCF-2 on growth over the course of the study including the one-month follow-up. Receipt of MDCF-2 was linked to the magnitude of change in levels of 70 β-WLZ-positively correlated plasma proteins including mediators of bone growth, neurodevelopment and inflammation (gene set enrichment analysis [GSEA];p<0.001) and the abundances of 23 WLZ-associated bacterial taxa (GSEA;p<0.001).

**CONCLUSIONS:**

These findings provide support for further clinical investigation of MDCF-2 as a dietary supplement for young children with MAM and provide insight into mechanisms by which this targeted manipulation of microbiota components may be linked to growth. (Supported by the Bill and Melinda Gates Foundation and the NIH; ClinicalTrials.gov identifier:NCT04015999)

Childhood undernutrition is a global health challenge that produces impaired ponderal and linear growth (wasting and stunting), immune and metabolic dysfunction, altered central nervous system (CNS) development as well as other abnormalities(1,2). Acute malnutrition in children is classified by their degree of wasting: e.g., moderate acute malnutrition (MAM) and severe acute malnutrition (SAM) are defined by a weight-for-length Z score (WLZ) that is 2–3 or >3 standard deviations, respectively, below the median of a WHO Multicentre Growth Reference Study cohort(3). Children with MAM and SAM have defects in the development of their gut microbiota, leaving them with microbial communities that appear younger than those of their healthy counterparts(4,5). Current nutritional interventions for MAM and SAM have not focused on the microbiota as a therapeutic target. Coincidentally, existing therapies have limited efficacy in treating the long-term sequelae that affect undernourished children(6,7) and in repairing their microbiota(8). With the COVID-19 pandemic estimated to increase childhood deaths from wasting by more than 20%(9), surmounting this already formidable global health challenge has become even more pressing.

We previously identified a network (‘ecogroup’) of 15 bacterial taxa whose covarying representation describes normal gut microbial community development during the first 2 years of postnatal life in healthy members of birth cohorts from several low- and middle-income countries(5). Changes in the abundances of ecogroup taxa provide a way of defining the severity of microbiota perturbations in children with untreated MAM and SAM, as well as characterizing the incomplete nature of microbiota repair that occurs when they receive existing therapeutic foods(5,8). Comparisons of gnotobiotic mice colonized with fecal microbiota from age-matched healthy children or those with wasting/stunting have revealed bacteria discriminatory for weight gain, a number of which are ecogroup taxa(5,10). Supplementing mice harboring microbiota from a wasted/stunted child with five of these strains prevented microbiota-dependent transmission of an impaired weight-gain phenotype(10). Based on these observations, and through screening combinations of food staples in gnotobiotic mice and gnotobiotic piglets, we developed several microbiota-directed complementary food (MDCF) prototypes(8). Three of these formulations were compared to an existing ready-to-use supplementary food (RUSF) in a 1-month-long, randomized controlled trial of 12–18-month-old children with MAM living in an urban slum in the Mirpur district of Dhaka, Bangladesh. One of these formulations (‘MDCF-2’) changed the microbiota to a composition similar to that of aged-matched healthy Mirpur children and changed the abundances of plasma proteins indicative of improved health status(5,8). We have now performed a larger, longer study to compare the effects of MDCF-2 and RUSF on clinical endpoints.

## METHODS

### Study design

This study was approved by the Ethical Review Committee at the icddr,b and conducted in Mirpur between November 2018 and December 2019. Parents/guardians of all study participants provided written informed consent.

**Fig. 1:**
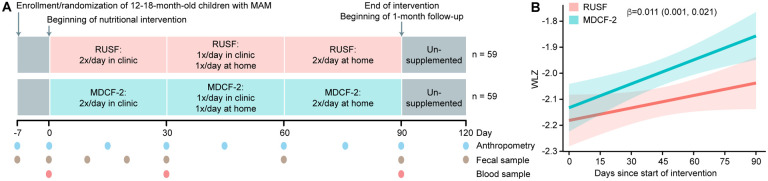
Study design of a randomized controlled trial of MDCF-2 or RUSF supplementation in children with MAM. **(A)** Study design. **(B)** WLZ during treatment. Best-fit linear regression lines are colored green (MDCF-2) or red (RUSF), and the lighter shaded areas around the lines indicate 95% confidence bands. The β-coefficient of +0.011 represents the weekly change in WLZ in children receiving MDCF-2 compared to those receiving RUSF. The 95% confidence interval is shown in parentheses.

Male and female children with MAM between 12- and 18-months-old who satisfied the inclusion/exclusion criteria (n=124) were randomized to receive MDCF-2 or RUSF (see Table S1 for compositions and nutritional analysis, Supplementary Methods for inclusion criteria and sample size calculation, and Figure 1A for study design and data/biospecimen collection schedules). The caloric density of MDCF-2 is lower than RUSF (204 versus 247 kcal/50 g daily dose, Table S1). Anthropometry was measured every 15 days while morbidity data were documented daily. Field Research Assistants monitored participants for any side effects/adverse events and treated them according to standard-of-care, if needed.

During the first month, two daily 25g servings of MDCF-2 or RUSF were provided to each child by her/his mother at a local study center under the supervision of a health-care provider; the amount unconsumed was determined by weighing. In the second month, one of the two daily feedings, and in the third month both daily feedings occurred at home under the supervision of a visiting health-care provider, with documentation of the amount consumed. Other than being asked to avoid feeding their children during the 2-hour period before each visit, mothers were advised to continue their usual breastfeeding and complementary feeding practices throughout the study. After completing three months of intervention, children returned to their normal feeding routine but continued to be monitored, with fecal samples and anthropometric data collected 1-month post-treatment.

### End points

Outcome measures were the weekly rate of change in weight-for-length z-score (WLZ), weight-for-age z-score (WAZ), mid-upper-arm circumference (MUAC), length-for-age z-score (LAZ), morbidity, plasma proteomic profile, and gut microbiota configuration.

### Statistical analysis

Changes in ponderal growth between treatment arms were compared using linear mixed-effects models that included a random-effect coefficient to control for differences in characteristics between individuals; other anthropometric measures were assessed in a similar fashion. Analyses of food-frequency questionnaire and morbidity data were performed using generalized linear mixed-effects models. Because we tested the effects of the supplements on four measures of growth and did not correct for multiple testing, we have provided confidence intervals for each outcome.

Changes in plasma protein abundances were analyzed using an Empirical Bayes linear model framework [limma(12)] and gene set enrichment analysis [GSEA(13)], a method for quantifying whether a rank-ordered list of features (e.g., proteins ranked by their changes in abundances after a treatment or by correlation coefficient) are enriched for a subset of features of interest (e.g., a biological pathway). Effects of supplementation on microbial community configuration were quantified using linear mixed-effects models and GSEA. For all statistical analyses, p<0.05 was considered as ‘statistically significant’. For analyses requiring adjustment for multiple hypotheses, those that reached a false discovery-rate (FDR) adjusted p-value (q-value) <0.1 or ≤0.05 for plasma proteomic and fecal microbial datasets, respectively, were deemed statistically significant. All reported p-values are two-sided.

## RESULTS

### Clinical characteristics and response to nutritional intervention

Children with MAM, 15.4±2.0 (mean±SD) months-of-age, living in Mirpur were enrolled in a 3-month randomized study of twice-daily dietary supplementation with either MDCF-2 (n=61) or RUSF (n=62) (Figure S1). Fifty-nine children in each arm completed the 3-month intervention and 1-month follow-up. Five did not complete the study, due to the family moving or withdrawal of consent (Figure S1).

At enrollment, anthropometric and socio-demographic characteristics did not differ between children in the two arms (Table 1, Table 2, Table S3). Over the 3-month intervention period, there was no difference in the amount of supplement consumed between children receiving MDCF-2 or RUSF [92.5±0.73% (mean±SEM) of the amount provided versus 92±1.15%, respectively; p=0.87]. There were no discernible differences in the proportion of children meeting WHO requirements for minimum meal frequency or minimum acceptable diet (Supplementary Results, see Table S4 for dietary practices).

A mixed effects linear model predicting anthropometry as a function of the interaction between treatment group and weeks since starting nutritional supplementation (controlling for the main effects of baseline age, gender, illness 7 days prior to starting the intervention, weeks in the intervention, treatment group, and a random intercept for each participant) yielded β-indices of 0.011 (CI, 0.001, 0.021) for WLZ, 0.008 (0.001, 0.015) for WAZ, 0.003 (CI, −0.001 , 0.007) for MUAC, and −0.001 (CI, −0.005, 0.003) for LAZ (Table 2) (A positive value indicates a faster growth rate in children receiving MDCF-2 compared to RUSF. For example, extrapolating a β-WLZ of +0.011 over a year period would equate to a gain of 0.57 WLZ compared to RUSF). β-indices for the 4-month period encompassing the 3-month intervention and 1-month follow-up were 0.010 (0.002, 0.018) for WLZ, 0.008 (0.002, 0.013) for WAZ, 0.004 (0.000, 0.007) for MUAC, and 0.000 (−0.003, 0.003) for LAZ (Figure 1B, Table 2, Table S5). See Figures S2, S3, Table S4 and Table S6) for effects on morbidity and dietary habits.

### Effects of Intervention on Plasma Proteome

**Fig. 2:**
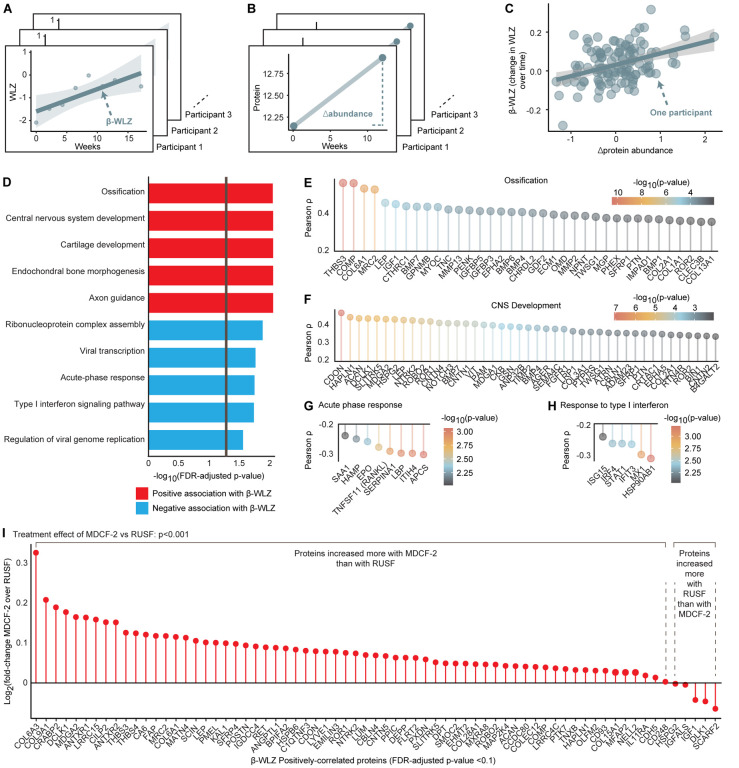
Effects of Nutritional Intervention on Plasma Proteins. **(A-C)** Schematic depicting the calculation of ‘β-WLZ’ for each participant (panel A), ‘Δprotein abundance’ for each participant (panel B) and the correlation between these two values (panel C). **(D)** Gene set enrichment analysis (GSEA) of proteins whose abundances were correlated with ponderal growth. The vertical gray line indicates q<0.05. **(E-H)** Gene Ontology (GO) terms enriched for by the set of WLZ-associated proteins. Shown are the correlation strengths between proteins belonging to a GO term and ponderal growth. Only proteins whose correlations with β-WLZ reached an unadjusted p<0.01 are shown. Proteins are ordered by correlation strength and colored by their p-value (transformed to a −log_10_ scale so that decreasing values indicate less statistical significance). **(I)** Differential effects of MDCF-2 and RUSF on WLZ-associated proteins. Proteins are ordered by the log_2_(fold-change) of the treatment effect of MDCF-2 over RUSF after three months of supplementation. GSEA was used to calculate the enrichment of proteins whose abundances were increased more by MDCF-2 compared to RUSF for the set of 70 proteins that are positively correlated with β-WLZ.

We quantified the abundances of 4,977 proteins(14) in plasma samples collected from all 118 children in the study at the 0-, 1- and 3-month time points (Table S2, Figure S4A,B). For each child, a linear model relating the duration of supplementation to his/her β-WLZ during the 3-month intervention was constructed; β-WLZ was then correlated with changes in the abundances of plasma proteins prior to and after completing the intervention (Δprotein abundance, Figure 2A–C). Seventy-five proteins were identified as significantly correlated (positively or negatively) with β-WLZ [false discovery rate (FDR)-adjusted q<0.1, Table S7]. Gene set enrichment analysis (GSEA) querying Gene Ontology (GO) ‘biological processes’ revealed that proteins positively correlated with β-WLZ were significantly enriched (q<0.1) for (i) mediators of bone growth and ossification [e.g., insulin-like growth factor 1 (IGF1); cartilage oligomeric matrix protein (COMP, an extracellular matrix protein critical for endochondral bone growth;^15^,^16^); secreted frizzled-related protein 4 (SFRP4, Wnt inhibitor that prevents excessive osteoclast erosion of bone;^17^) (Figure 2D,E; Table S7,S8)] and (ii) CNS development [e.g., the axon guidance protein SLIT and NTRK-like protein 5 (SLITRK5); BDNF/NT-3 growth factor receptor (NTRK3) and roundabout homolog 2 (ROBO2, axon guidance receptor with pro-osteoblastic/anti-osteoclastic activities;^18^) (Figure 2F, Table S7,S8)]. Proteins whose abundances negatively correlated with ponderal growth were significantly enriched for acute phase reactants and actuators of immune activation [e.g., hepcidin (HAMP; reduces iron absorption and induces iron sequestration during inflammatory states); RANKL (osteoclast-promoting factor); granulysin (GNLY, proinflammatory cytokine produced by activated cytotoxic T- and NK-cells); interferon-induced protein with tetratricopeptide repeat-3 (IFIT3, inhibits the replication of multiple viral pathogens(19)); and immunoglobulin A (IGHA1) (Figure 2G,H, Table S7,S8)].

A total of 714 proteins exhibited significantly (q<0.1) higher or lower levels after the 3-month MDCF-2 supplementation, in contrast with 82 proteins that showed significant alterations after RUSF treatment (Table S9A,B). Proteins that showed increases after 3 months of supplementation with MDCF-2 were significantly enriched for the 70 positively β-WLZ-associated proteins (GSEA p<0.001), in contrast with the proteins that showed increases after RUSF supplementation (GSEA p=0.11, Figure S5A,B). Comparing the two treatments revealed that proteins whose levels increased more with MDCF-2 were significantly enriched for WLZ-associated proteins (GSEA p<0.001, Figure 2I). Of the WLZ-associated proteins elevated after MDCF-2 supplementation, cartilage intermediate layer protein 2 (CILP2) was elevated to the greatest extent. Its levels did not change in children who received RUSF. It forms complexes with collagen VI to promote articular cartilage formation and regulates metabolic status(20). Other proteins significantly increased by MDCF-2 but not RUSF included thrombospondin-4 (THBS4), a multifunctional protein involved in bone, skeletal muscle, vascular, and nervous system development(21), and the osteoclast inhibitor SFRP4.

Analysis of the plasma proteomes of children given MDCF-2 by quartile of ponderal growth rate (β-WLZ) (n=15 children/group) revealed that those in the upper quartile started off more wasted, were more deplete of mediators of bone growth and neurodevelopment and had higher levels of pro-inflammatory proteins compared to those in the lower quartile. By the end of MDCF-2 supplementation, children in the upper quartile manifested the largest increases in mediators of bone growth and CNS development and the largest decreases in effectors of inflammation (Figure S6A,B, Tables S10,S11; Supplementary Results). Together, these results provide evidence that mediators of bone growth, neurodevelopment and inflammation distinguish the effects of the microbiota-directed nutritional intervention from that of RUSF.

### Effects of Supplementation on Gut Microbiota

To determine the effects of supplementation on gut microbiota, fecal samples were obtained every 10 days during the first month of the intervention and monthly thereafter. Quantitative PCR assays revealed no statistically significant differences between treatments in the representation of 23 bacterial, viral and protozoan enteropathogens (Table S12). A more comprehensive analysis was obtained by identifying bacterial taxa through sequencing of PCR amplicons generated from variable region 4 of 16S rDNA genes present in fecal biospecimens (Figure S7). We then used linear mixed-effects models to determine the relationships between the abundances of bacterial taxa [defined by the representation of amplicon sequence variants (ASVs); see Supplementary Methods] and WLZ in each participant.

**Fig. 3:**
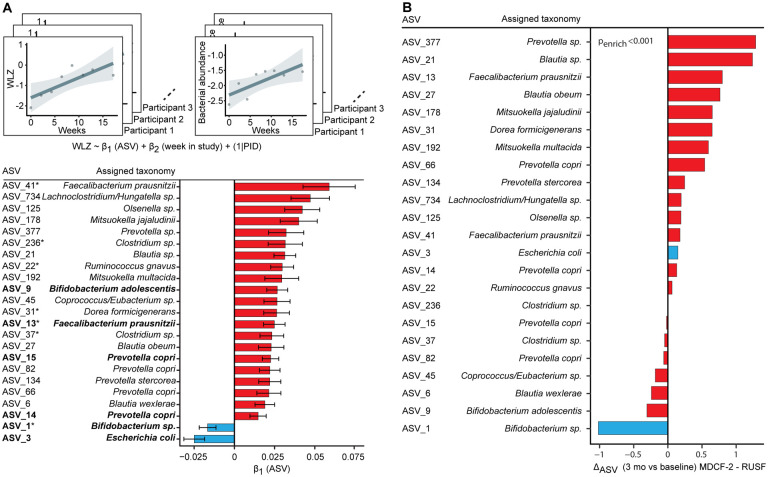
Response of the Gut Microbiota to MDCF-2 and RUSF Supplementation. **(A)** Analytical scheme for linear mixed effects modeling of the relationship between WLZ and taxon (ASV) abundance during supplementation. The coefficient β_1_ represents the change in WLZ for a unit change in the variance-stabilizing, transformed abundance of an ASV. A random effect for participant ID (PID) was included in the model to account for repeated measurements taken from the same individual. Bar graphs indicate β_1_ (the linear model coefficients) ± SEM for each taxon that was significantly associated with WLZ. ASVs in bold-face were previously identified as ‘ecogroup’ taxa(5), while those with asterisks have previously described associations with weight gain in gnotobiotic mice harboring gut microbial communities obtained from healthy and undernourished children(10). **(B)** Ratio of 3-month Δ_ASV_ (the change in variance-stabilizing transformed ASV counts prior to and after supplementation) between MDCF-2 and RUSF treatment arms. A positive ratio indicates a greater average increase in children treated with MDCF-2. Color scheme: red bars, ASVs with significant positive associations with WLZ; blue bars, ASVs with significant negative associations with WLZ.

Analyzing ASV data generated from 706 fecal samples with temporally-matched anthropometry from all participants revealed that among the 209 ASVs that satisfied our threshold for prevalence and abundance, 23 were significantly correlated with WLZ (‘WLZ-associated’ taxa); 21 were positively associated while two were negatively associated (*Bifidobacterium* sp. (ASV_1, likely *B. longum*) and *Escherichia coli* (ASV_3) (Figure 3A, Table S13). Six of the WLZ-associated taxa (bolded ASVs in Figure 3A) are members of the ecogroup network of bacteria whose representation describes normal gut microbial community development in healthy Bangladeshi infants/children(5). In this previous study, microbiota repair by MDCF-2 was accompanied by changes in two of these six WLZ-correlated ecogroup taxa: increases in *Prevotella copri* and reductions in *Bifidobacterium longum*(5). Five of the WLZ-associated ASVs identified in the present study (asterisks in Figure 3A) share taxonomic similarity with bacteria identified as discriminatory for weight gain in our previous studies of gnotobiotic mice colonized with fecal microbiota from healthy versus malnourished children; they include *Faecalibacterium prausnitzii*, *Dorea formicigenerans*, *Ruminococcus gnavus* and a member of *Clostridium.* Importantly, the complementary food ingredients incorporated into MDCF-2 were selected based on their abilities to increase the fitness and expressed beneficial functions of these age- and growth-discriminatory taxa(8). An analysis of covariance between features of the plasma proteome and members of the gut microbiota revealed that the abundances of these 21 WLZ-positively associated taxa were associated with the 70 plasma proteins whose levels positively correlated with β-WLZ (Figures S8–S10; Tables S14, S15; Supplementary Results). See Supplementary Results and Table S16 for analyses of bacterial taxa associations with dietary practices.

The abundances of WLZ-associated taxa were significantly greater in the gut microbiota of children whose diets were supplemented by MDCF-2 than in those of children whose diets were supplemented by RUSF (p<0.001, GSEA; Figure 3B). Figure S11 and Table S17 rank WLZ-associated taxa based on the magnitude of their changes in relative abundances during MDCF-2 treatment; the greatest increases occurred with *P. copri* and *F. prausnitzii,* while the *Bifidobacterium* sp. (likely *B. longum*) exhibited the greatest decrease. Additionally, compared to (unsupplemented) children from Mirpur with consistently healthy growth phenotypes (WLZ ≥−1) and normally developing microbiota, MDCF-2 produced a significant enhancement in the rate of change in abundances of ASVs assigned to members of *Prevotella* (including *P. copri*), *F. prausnitzii*, *Olsenella*, and *Bifidobacterium* (*B. longum*) – i.e. those among the most highly positively and negatively correlated with WLZ. In contrast, RUSF did not elicit significant differences in the rate of change in abundance of any of the 23 WLZ-correlated ASVs (Figure S12, Table S18, S19). One month after cessation of supplementation, levels of a majority of MDCF-2-responsive, WLZ-associated ASVs had begun to fall at the same time that WLZ was diminishing (Figure S13A–D).

## DISCUSSION

We describe the results of a randomized, controlled feeding study testing the effects of a microbiota-directed complementary food (MDCF-2) against an existing supplementary food (RUSF) on four measures of growth in 12–18-month-old Bangladeshi children with MAM. The rate of changes we observed in WLZ score, WAZ score and MUAC, but not LAZ score, support a benefit of MDCF-2 on growth despite its lower caloric density compared to RUSF. We observed larger changes in plasma protein mediators of bone growth, neurodevelopment and inflammation and more complete repair of the gut microbiota of children who received MDCF-2 compared with those who received RUSF.

We did not attempt to test the effects of MDCF-2 and RUSF on body composition (changes in fat-versus lean-mass). Chronic undernutrition in early life induces metabolic reprogramming that may enable a child to more efficiently capture and store energy as fat during periods of nutrient scarcity(22). While adaptive in the short-term, this metabolic shift predisposes children to developing diabetes, hypertension, and cardiovascular disease later in life(23). MDCF-2 elicits a concerted change in WLZ-associated proteins, a number of which are effectors of bone growth and skeletal muscle development. Some of these proteins have also been implicated in metabolic disorders(24–30). However, augmenting growth of bone and skeletal muscle may promote a rebalancing of the rapid ‘catch-up’ fat accretion, observed when undernourished children are given standard nutritional interventions, towards a more appropriate lean-to-fat mass ratio, simultaneously improving growth and protecting from later obesity(31,32).

Studies conducted in children with MAM in Malawi and Ethiopia indicate that increases in ‘fat-free’ mass accretion in the first two years of life are associated with better cognitive and motor development(33,34). Children who received MDCF-2 exhibited increased plasma levels of proteins associated with axonal growth and CNS development, but whether this observation has anything to do with changes in the central nervous system is not known. It will be important to follow cohorts of children with MAM treated with MDCF-2 for longer periods of time to assess whether the observed changes in these proteins correlate with clinical improvements in cognitive performance.

A hallmark of a successfully executed program of gut community development in Bangladeshi children (and those in other resource-poor settings) is the transition from a *Bifidobacterium longum*-dominated community during exclusive/predominant breastfeeding, to one in which *Prevotella copri* becomes a major component during weaning(5). While *B. longum* has been associated with numerous beneficial outcomes in breast-feeding infants, its abundance was negatively associated with ponderal growth rate in the 12–18-month-old children enrolled in the present study, which underscores the importance of considering how the timing of nutritional intervention aligns with the state of microbiota development.

Larger trials performed in disparate geographies are needed to further assess the efficacy of this therapeutic approach for treating childhood undernutrition. The plasma and microbiota biomarkers identified in the present study should help enable better characterization (and stratification) of participants in future interventions.

## Figures and Tables

**Table 1:** Clinical characteristics at enrollment

	MDCF-2 (n=61)	RUSF (n=62)
**Demographic Features**		
Age (months)	15.4 ± 1.9	15.5 ± 2.0
Female - no. (%)	35 (57%)	36 (58%)
**Anthropometry at enrollment**		
WLZ	−2.31 ± 0.29	−2.40 ± 0.27
WAZ	−2.69 ± 0.67	−2.76 ± 0.62
LAZ	−2.08 ± 1.16	−2.08 ± 1.12
MUAC (cm)	12.8 ± 0.53	12.7 ± 0.44
**Breastfeeding status†**		
Not breastfed since birth - no. (%)	1 (2%)	0 (0%)
Partial breastfeeding - no. (%)	46 (75%)	46 (74%)
Exclusive breastfeeding - no. (%)	14 (23%)	16 (26%)
**Immunization status**		
Complete - no. (%)	53 (87%)	52 (84%)
Partial - no. (%)	8 (13%)	6 (10%)
None - no. (%)	0 (0%)	4 (6%)

WLZ: weight-for-length z-score. WAZ: weight-for-age z-score. LAZ: length-for-age z-score. MUAC: mid-upper arm circumference.

Values represent: mean ± SD; number (%); median [interquartile range].

**Table 2:** Clinical response to MDCF-2 or RUSF supplementation

Anthropometry at the start of intervention i.e. baseline			
	MDCF-2 (n=59)†	RUSF (n=59)†	β Treatment (95% CI)‡
WLZ	−2.22 ± 0.39	−2.29 ± 0.36	0.086 (−0.056, 0.228)
WAZ	−2.66 ± 0.67	−2.71 ± 0.64	0.036 (−0.213, 0.285)
LAZ	−2.14 ± 1.14	−2.13 ± 1.13	−0.044 (−0.467, 0.380)
MUAC (cm)	12.8 ± 0.51	12.7 ± 0.44	0.077 (−0.100, 0.254)
Rate of growth during intervention (Δ anthropometry/week)			
	β growth rate for MDCF-2 (95% CI)§	β growth rate for RUSF (95% CI)§	β Interaction (95% CI)¤
WLZ	0.021 (0.014, 0.029)	0.010 (0.003, 0.017)	0.011 (0.001, 0.021)
WAZ	0.017 (0.012, 0.022)	0.010 (0.004, 0.015)	0.008 (0.001, 0.015)
LAZ	0.004 (0.002, 0.007)	0.005 (0.003, 0.008)	−0.001 (−0.005, 0.003)
MUAC (cm)	0.031 (0.029, 0.034)	0.029 (0.025, 0.032)	0.003 (−0.001, 0.007)
Rate of growth including the 1-month follow-up (Δ anthropometry/week)			
	β growth rate for MDCF-2 (95% CI)§	β growth rate for RUSF (95% CI)§	β Interaction (95% CI)¤
WLZ	0.010 (0.005, 0.016)	0.000 (−0.005, 0.006)	0.010 (0.002, 0.018)
WAZ	0.009 (0.005, 0.013)	0.001 (−0.003, 0.005)	0.008 (0.002, 0.013)
LAZ	0.004 (0.002, 0.006)	0.003 (0.001, 0.006)	0.000 (−0.003, 0.003)
MUAC (cm)	0.028 (0.026, 0.031)	0.024 (0.022, 0.027)	0.004 (0.000, 0.007)

† Values represent the mean ± SD.

‡ Linear model predicting anthropometry at the start of the intervention as a function of treatment group, controlling for baseline age, gender and any illness 7 days prior to starting the intervention. β indicates the mean difference in anthropometry between participants who were assigned to the MDCF-2 and RUSF arms at the start of the intervention. CI, confidence interval.

§ Mixed effects linear model predicting anthropometry as a function of weeks since starting nutritional supplementation, controlling for the main effects of baseline age, gender, any illness 7 days prior to starting the intervention, and a random intercept for each participant. β indicates the growth rate in unit (anthropometric measure) per week.

¤ Mixed effects linear model predicting anthropometry as a function of the interaction between treatment group and weeks since starting nutritional supplementation, controlling for the main effects of baseline age, gender, any illness 7 days prior to starting the intervention, weeks in the intervention, treatment group, and a random intercept for each participant. β indicates the interaction between treatment and growth rate in unit/week (a positive value indicates a faster growth rate in children receiving MDCF-2).
