# Supplementary material for: A Microbiota-Directed Food Intervention for Undernourished Children
Source: N Engl J Med. Author manuscript; Available in PMC 2021 Apr 8. (PMC7993600; doi:10.1056/NEJMoa2023294)
Supplement: Supplementary file 1 [file NEJMoa-2021-2023294-s1.pdf]

ONLINE SUPPLEMENT

TABLE OF CONTENTS

Page

List of Investigators 5

SUPPLEMENTARY METHODS 6

Nutritional supplementation 6

Sample collection 7

Immunization and breastfeeding status 8

Preparation of MDCF-2 and RUSF 8

Processing of plasma samples 9

Processing of fecal samples for microbiota analysis 10

Quantification of 23 enteropathogens by multiplex qPCR 11

STATISTICAL ANALYSIS 11

Study design: power calculation 11

Analysis of enrollment and baseline characteristics 11

Analysis of anthropometric responses to MDCF-2 or RUSF 12

Robust linear mixed-effects modeling 13

Analysis of illness and co-morbidities during supplementation 13

Analysis of dietary habits during supplementation 14

Identification of 'WLZ-associated' proteins 15

Differential abundance analysis of plasma proteins 16

Analysis of gut microbiota reconfiguration 17

Determining associations between food frequency questionnaire responses and ASV abundances 18

Negative binomial singular value decomposition (NB-SVD) analysis 18

DATA DEPOSITION 20

SUPPLEMENTARY RESULTS 21

Effects of MDCF-2 and RUSF on mid-upper arm circumference (MUAC)........................... 21

Effects of MDCF-2 and RUSF on co-morbidities.. 21

Changes in dietary habits during MDCF-2 and RUSF supplementation 21

Associations between food frequency questionnaire responses and ASV abundances........... 22

Relating features of the plasma proteome to members of the gut microbiota......................... 23

MDCF-2 responsiveness and durability of response... 24

SUPPLEMENTARY FIGURES 27

Figure S1. Study summary 28

Figure S2. Effects of MDCF-2 and RUSF on illness and co-morbidities 29

Figure S3. Food frequency questionnaire responses as a function of treatment with MDCF-2 or RUSF ……………………. 30

Figure S4. Quality control of proteins represented on the SomaScan platform.................................. 32

Figure S5. Effects of MDCF-2 and RUSF on WLZ-associated proteins 33

Figure S6. Analysis of MDCF-2 responsiveness.. 34

Figure S7. Analysis of V4-16S rDNA amplicon sequencing depth 35

Figure S8. Method for relating features of the plasma proteome to members of the gut microbiota... 36

Figure S9. Results from negative binomial singular value decomposition analysis 38

Figure S10. Complete SV8 cross-association profile identified by NB-SVD analysis........................ 39

Figure S11. Effects of supplementation on WLZ-associated taxa 41

Figure S12. Comparing the rate of change in representation of WLZ-correlated ASVs in the gut microbiota of healthy Mirpur children with their representation in similarly-aged children with MAM receiving MDCF-2... 42

Figure S13. Determinants and predictors of MDCF-2 responsiveness 43

SUPPLEMENTARY TABLES 45

Table S1. Nutritional composition of MDCF-2 and RUSF... 45

Table S2. Ledger of enrollment characteristics and samples obtained from children in the study...... 45

Table S3. Environmental characteristics at enrollment 45

Table S4. Food frequency questionnaire responses during the 3-month period of treatment 45

Table S5. Robust linear mixed-effects modeling analyses of anthropometric response to MDCF-2 versus RUSF supplementation 46

Table S6. Effects of supplementation on co-morbidity and illness 47

Table S7. Correlations between changes in protein abundances and ponderal growth. 47

Table S8. Gene set enrichment analysis of WLZ-associated proteins 47

Table S9. Differential abundance analyses of the plasma proteomes of children undergoing nutritional supplementation 47

Table S10. Differential abundance analysis of the plasma proteomes between upper- and lower-quartile MDCF-2 β-WLZ responders 48

Table S11. Gene set enrichment analysis of differentially abundant plasma proteins between MDCF-2 upper-quartile and lower-quartile responders 48

Table S12. qPCR-based assays of enteropathogens 48

Table S13. Associations between changes in the abundances of bacterial taxa (ASVs) and rates of ponderal growth 48

Table S14. Projections along the first 10 singular vectors (SVs) identified from NB-SVD analysis .. 48

Table S15. Enrichment of singular vector 8 protein projections for GO ‘biological processes’ 49

Table S16. Associations between food frequency questionnaire responses and abundances of WLZ-correlated ASVs 49

Table S17. Changes in abundances of bacterial taxa in children undergoing MDCF-2 or RUSF supplementation 49

Table S18. Linear mixed-effects modeling analysis of gut microbial configuration in healthy children or children with MAM treated with MDCF-2 or RUSF 49

Table S19. Associations between baseline ASV abundances and β-WLZ in children who received

MDCF-2 49

Table S20. Changes in the abundances of ASVs after MDCF-2 supplementation between upper-quartile versus lower-quartile β-WLZ responders 49

SUPPLEMENTARY REFERENCES 50

List of Investigators

Robert Y. Chen, B.S.[1,2]*, Ishita Mostafa, B.D.S., M.P.H.[3]*, Matthew C. Hibberd, Ph.D.[1,2,4]*, Subhasish Das, M.B.B.S., M.P.H.[3], Mustafa Mahfuz, M.B.B.S., M.P.H.[3], Nurun N. Naila, M.B.B.S., M.P.H.[3], Md.Munirul Islam, M.B.B.S., Ph.D.[3], Sayeeda Huq, M.B.B.S., M.P.H.[3], Md.Ashraful Alam, M.P.H.[3], Mahabub Uz Zaman, M.P.H.[3], Arjun S. Raman, M.D., Ph.D.[1,2,4], Daniel Webber, M.D., Ph.D.[1,2,4], Cyrus Zhou, B.S.[1,2], Vinaik Sundaresan, B.S.[1,2], Kazi Ahsan, M.B.B.S., M.P.H.[1,2], Martin F. Meier, B.S.[1,2], Michael J. Barratt, Ph.D.[1,2,4], Tahmeed Ahmed, M.B.B.S., Ph.D.[3#], Jeffrey I. Gordon, M.D.[1,2,4#+].

[*]Contributed equally; [#]co-senior authors

[1]Edison Family Center for Genome Sciences and Systems Biology, Washington University School of Medicine, St. Louis, MO 63110 USA

[2]Center for Gut Microbiome and Nutrition Research, Washington University School of Medicine, St. Louis, MO 63110 USA

[3]International Centre for Diarrhoeal Disease Research, Bangladesh (icddr,b), Dhaka 1212, Bangladesh

[4]Department of Pathology and Immunology, Washington University in St. Louis School of Medicine, St Louis, MO 63110 USA.

SUPPLEMENTARY METHODS

Nutritional supplementation

During the first month of the study, each child was brought to a study center twice daily (morning and afternoon). On each visit, mothers were provided 25 g of their assigned food supplement (MDCF-2 or RUSF) and asked to spoon feed their child, under the supervision of trained study personnel, until she/he refused to eat further. The amount of food consumed at each visit was recorded by subtracting that left over from the offered amount (pre-weighed napkins were used to collect any food regurgitated or spilled, which was deducted from the amount provided). In the second month, each child was provided 25 g of their assigned food supplement at the feeding center, and an additional 25 g was provided in a clean container to feed at home that afternoon. In the third month, two separate containers (for morning and afternoon feeding) containing 25 g of study diet were delivered each day to each enrolled child at her/his home. Any unconsumed diet from each feeding was retained in the container; the weight of study diet consumed was determined by weighing the food remaining in the container. Mothers were advised to avoid feeding their children during the 2-hour period before each visit but to otherwise continue their usual breastfeeding and complementary feeding practices throughout the day/study.

Current recommendations for treating children with MAM are predicated on the principle that dietary management should be based on the optimal use of locally available nutrient-dense foods to improve their nutritional status and prevent them from becoming further malnourished (e.g., developing SAM). However, at present, there are no evidence-informed recommendations regarding the composition of supplementary foods used to treat children with MAM (*1*,*2*).

To develop MDCFs, we selected 12 locally available complementary food ingredients that are commonly consumed by the target population in Dhaka, Bangladesh. We designed 21 unique diets containing different combinations of these ingredients and tested them in gnotobiotic mice colonized with a consortium of 9 ‘target’ bacterial strains; these strains were selected based on their underrepresentation in the fecal microbiota of Bangladeshi children with acute malnutrition when compared to the microbiota of age-matched healthy children from the same population. (Note that administration of a consortium of

five of these target strains had been previously shown to ameliorate growth faltering in gnotobiotic mice colonized with intact microbiota from a stunted, underweight child) (*3*). We identified ingredients (chickpea, peanut, banana and soyflour) that significantly promoted the representation of one or more of the target bacterial strains. Formulations containing these ingredients were subsequently tested in gnotobiotic piglets colonized with the bacterial targets to ascertain their effects on bacterial fitness as well as on host biology in a species that is physiologically and metabolically more similar to humans than mice. Based on these results and tests of organoleptic acceptability, lead MDCF prototypes were advanced to a 1-month controlled feeding study involving 15-17 Bangladeshi children with MAM/arm to determine their capacity to produce microbiota repair. This study led to the selection of MDCF-2 for the follow-up POC trial described here due to the superiority of its ability to promote microbiota repair and its beneficial effects on plasma biomarkers of healthy growth.

In addition to the inclusion of microbiota-directed ingredients, MDCF-2 was designed to approximate the recommended nutritional composition of specially formulated foods for the prevention and treatment of acute malnutrition in 12-18 month-old children (*2*); specifically, provision of ~250 kcal/day of supplemental energy, of which 45–50 percent should be from fat and 8–10 percent from protein. Culturally acceptable, ready-to-use supplementary foods (RUSF) composed of locally-available food ingredients (rice/lentils) have been previously demonstrated to provide modest but statistically significant improvements in linear growth (compared to nutritional counselling alone) after 12 months of treatment of rural Bangladeshi children during the weaning period (6-18 months) (*4*,*5*). A recent meta-analysis indicated that supplementary foods are often more effective at promoting anthropometric recovery in children with MAM than nutrition counseling, with or without the addition of micronutrient supplements. In addition, supplementation with high-quality protein and adequate micronutrient content, for 3 months, is recommended (*6*).

Sample collection

Fecal samples were collected at participants' homes within 20 minutes of production by study personnel, transferred in 2 mL cryovials to Cryo Exchange vapor shippers (Taylor-Wharton/Worthington Industries, CX-100) and transported to the study center where they were recorded and stored at -80 ^{o}C. EDTA-plasma was prepared from blood collected during scheduled visits to the study center as previously described (*7*) and stored at -80 ^{o}C. Coded biospecimens were shipped to Washington University on dry ice where they were stored at -80 ^{o}C, along with associated metadata, in a dedicated repository with approval from the Washington University Human Research Protection Office.

Immunization and breastfeeding status

In Bangladesh, the 'Expanded Programme on Immunization' (EPI) starts with Bacillus Calmette–Guerin (BCG) vaccine, pentavalent vaccine, inactivated polio vaccine (IPV) and bivalent oral poliovirus vaccine (bOPV), Pneumococcal conjugate vaccine (PCV) and measles and rubella (MR) virus vaccine to protect children from nine preventable diseases. We categorized immunization status as 'complete' (received all six vaccinations), 'partial' (completed less than six), and 'none' (not vaccinated at all). Breastfeeding status at enrollment was categorized as 'exclusively breast fed', 'partially breast fed' or 'never breast fed' since birth.

Preparation of MDCF-2 and RUSF

A food processing laboratory was established in the Mirpur area, in close proximity to the nutrition centers where the intervention was provided. All raw ingredients were purchased from a single local market in Dhaka. Each step of food preparation, including cleaning, roasting, particle size reduction, homogeneous blending, and supply to the nutrition centers was performed and monitored by icddr,b study investigators and field supervisors. Upon receiving the raw dry food ingredients (rice, lentils, chickpeas, soybeans, peanuts), any foreign material, grains or seeds were removed manually and by using a sieve. Ingredients were roasted in an open pan at 120-130 °C for 8-10 minutes, then allowed to cool and subsequently ground. At this stage, peanut was ready for mixing. The other food ingredients were converted into fine particles by blending for 4 to 5 minutes and sieving. Sugar was ground and the resulting fine powder was mixed with the other ingredients. Unpeeled whole green bananas were placed

in a deep pan and boiled in water for 17-20 minutes until they were tender. The peel was removed and the fruit was grated into small pieces which, after cooling, were mashed with a potato masher. The weights of all the ingredients required for preparing MDCF-2 and RUSF were recorded, pre-weighed micronutrient premix powder was added, and the supplementary foods were produced in small batches by mixing all ingredients in an electric blender.

The MDCF-2 and RUSF formulations were prepared fresh daily, dispensed and fed to participants on the same day. Samples of the food were routinely cultured at the icddr,b Food Safety Laboratory; tests included scoring total aerobes, total coliforms, *Escherichia coli*, *Enterobacteriaceae*, *Bacillus cereus*, *Salmonella* spp, *Shigella* spp, *Campylobacter* spp, coagulase positive and other *Staphylococci*, as well as yeasts and molds. The nutritional composition (energy content, moisture, protein, total fat, total carbohydrate, dietary fiber, ash) of the ingredients was assessed at the Institute of Nutrition, Mahidol University, Thailand using standard procedures.

Processing of plasma samples

The aptamer based SomaScan 5K Proteomic Assay plasma/serum kit (SomaLogic) was used to quantify the abundances of 5,284 proteins in plasma samples collected from children. Plasma samples were processed according to manufacturer's instructions as previously described (*8*). Briefly, 50 μL of plasma was incubated with NHS-biotin-tagged, protein-specific aptamer probes ('SOMAmers') to form protein-SOMAmer complexes that were immobilized on streptavidin beads. The complexes were subsequently cleaved, denatured, eluted, and hybridized to a custom Agilent DNA microarray. Arrays were scanned with an Agilent SureScan instrument at 5 μm resolution and the Cy3 fluorescence signal was quantified and processed using SomaLogic's SomaScan standardization procedures (*8*). SOMAmers that were not specific to human proteins, or that were marked by SOMAlogic as deprecated, were removed. Additionally, SOMAmers were removed whose median fluorescence signal across all samples was within 4.9 median average deviances (MAD) from blanks, resulting in a total of 4,977 SOMAmers that passed quality control (**Figure S4**). Protein abundances were $\log_2$-transformed and quantile-normalized prior to all downstream analyses (*8*).

Processing of fecal samples for microbiota analysis

Fecal samples were pulverized in liquid nitrogen. Methods for DNA extraction and purification have been described previously (*9*). Briefly, DNA was extracted from ~50 mg of pulverized material by bead-beating with 500 mL of 0.1 mm diameter zirconia/silica beads in a solution of 500 ml phenol:chloroform:isoamyl alcohol (25:24:1), 210 mL 20% SDS, and 500 mL buffer A (200 mM NaCl, 200 mM Trizma base, 20 mM EDTA), followed by purification (Qiaquick columns, Qiagen) and storage in Tris-EDTA (TE) buffer. Purified DNA was quantified (Quant-iT dsDNA broad range kit, Invitrogen), adjusted to 1 ng/uL concentration, and used to generate bacterial V4 16S rDNA amplicons and subsequent indexed Illumina libraries (*9*). Libraries were quantified, pooled, and sequenced using an Illumina MiSeq instrument to generate paired-end, 250 nt reads ($3.29 \times 10^4 \pm 9.93 \times 10^3$ reads/sample; mean ± SD). Amplicon sequences were processed to trim adapter and primer sequences using bbtools (v37.02). DADA2 (*10*) was used to analyze preprocessed, paired-end sequence data to obtain and quantify error-corrected amplicon sequence variants (ASVs) in R (v3.6.1). Taxonomic assignments were performed using the DADA2 implementation of the Ribosomal Database Project Naïve Bayesian Classifier (database v16) at a minimum bootstrap confidence of ≥ 80% (option 'minboot = 80'). Tables of ASV abundances (counts) for each sample were combined with sample metadata and taxonomic assignment into a phyloseq (v1.3.0) object in R. Samples with fewer than 2000 reads were excluded from further analysis. In addition to removing samples with insufficient absolute read depth, we also performed a rarefaction analysis based on the R package vegan (v2.5.6). Samples for which ASV richness reached saturation at their corresponding sampling depth were considered sufficiently sampled (**Figure S7**). Contaminating mitochondrial or chloroplast ASV sequences were removed, along with any bacterial-origin ASVs lacking phylum-level taxonomic classification. A count filter was applied to remove any ASVs present below five counts in fewer than 5% of samples, yielding a filtered table containing 209 ASVs across 939 samples. This filtered ASV table was adjusted for library size and normalized (variance stabilizing transformation) using DESeq2 (*11*).

Quantification of 23 enteropathogens by multiplex qPCR

Samples were prepared as described in (*9*). Briefly, DNA and RNA were extracted and purified from fecal samples. cDNAs were prepared using the High Capacity cDNA Reverse Transcription Kit (Applied Biosystems, Foster City, CA). The resulting cDNA products were amplified using Specific Target Amplification, hybridized with TaqMan primers and probes against 23 bacterial, viral, and protozoan pathogens (*12*) and quantified using the 96.96 Dynamic Array microfluidic digital PCR system (Fluidigm Corp. San Francisco, CA). The qPCR assay used in this study has been widely deployed in large multi-center cohort studies of undernourished children involving mixed-species microbial communities and is highly specific for the 23 target pathogens in these contexts (*13,14*).

STATISTICAL ANALYSIS

Study design: power calculation

In our previous 1-month-long, pilot study of different MDCFs (*9*), the pretreatment WLZ score of children assigned to the MDCF-2 arm was -2.2. After one month of supplementation, WLZ was -1.7. Considering a WLZ of -2 at baseline and -1.7 at the end of the intervention, and a pooled SD of 0.53, we calculated that a sample size of 49 in each arm would be necessary to achieve 80% power at a 5% level of significance. Furthermore, assuming a 20% rate of attrition, we determined that 62 children would be required per treatment group for the 4-month-long study described in the current report.

Analysis of enrollment and baseline characteristics

Comparisons of demographic, anthropometric, and environmental features at enrollment between children receiving MDCF-2 or RUSF were performed using two-sided unpaired t-tests for normally distributed features, Wilcoxon rank-sum tests for measurements with skewed distributions, or Chi-squared tests for categorical variables. The first day of intervention began on average 5.88 ± 0.14 (SEM) days after enrollment. For all analyses of anthropometric measurements, the first day of intervention was used as the baseline measurement. Comparisons of baseline anthropometric measures between children receiving

MDCF-2 and those receiving RUSF were performed using a linear model controlling for baseline age, gender, and any history of illness 7 days prior to enrollment.

Analysis of anthropometric responses to MDCF-2 or RUSF

Ponderal growth rate was calculated using a mixed-effects linear model that predicted WLZ from weeks in the intervention, controlling for baseline age, gender, any history of illness 7 days preceding enrollment, and a random intercept for each participant ID (PID). The model took the form:

$$WLZ \sim \beta_1(weeks\ in\ intervention) + \beta_2(baseline\ age) + \beta_3(gender) + \beta_4(history\ of\ illness) + (1|PID) \quad (1)$$

The rates of growth for children receiving MDCF-2 or RUSF reported in **Table 2** are β_1 in Equation (1) and represent the weekly increase in WLZ in a given treatment arm. The same equation was used to assess weekly improvements in WAZ, MUAC and LAZ.

A comparison of the effects of MDCF-2 and RUSF on growth rates was performed using a mixed effects linear model predicting WLZ from the interaction between weeks in the intervention and treatment, controlling for baseline age, gender, any history of illness 7 days preceding enrollment, weeks in the intervention, treatment, and a random intercept for each participant. The model took the form:

$$WLZ \sim \beta_1(treatment{:}\ weeks\ in\ intervention) + \beta_2(baseline\ age) + \beta_3(gender) + \beta_4(history\ of\ illness) + \beta_5(treatment) + \beta_6(weeks\ in\ intervention) + (1|PID) \quad (2)$$

The differential rate of ponderal growth as a function of treatment arm (MDCF-2 versus RUSF) reported in **Table 2** is β_1 in Equation (2) and represents how much more WLZ improved in children receiving MDCF-2 compared to those receiving RUSF per week. Equation (2) was also used to compare the effects

of MDCF-2 and RUSF on rates of change of WAZ, MUAC and LAZ by substituting WLZ for the anthropometric measure.

Robust linear mixed-effects modeling

A comparison of the effects of MDCF-2 versus RUSF was repeated using a robust linear mixed-effects modeling approach that controls for heavy-tailed distributions (i.e., outliers) attributable to both between- and within-subject variation (*15*). Equation (2) was used to estimate model coefficients using the rlmer function in the R package robustlmm v2.3 (*16*). The differential rate of growth as a function of treatment arm (MDCF-2 versus RUSF) reported in **Table S5** is β_1 in Equation (2), and represents how much more WLZ, WAZ, LAZ, or MUAC improved in children receiving MDCF-2 compared to those receiving RUSF per week. P-values were computed using the pt function in R, which calculates a p-value by drawing from the Student's t-distribution given a t-statistic (from rlmer) and an estimated degrees-of-freedom (approximated by the Welch-Satterthwaite equation).

Analysis of illness and co-morbidities during supplementation

For each intervention, the change in prevalence of cough, rhinorrhea, fever or diarrhea was quantified using a generalized mixed-effects linear model with a 'logit' link function that predicted a given co-morbidity from weeks in the intervention, controlling for a random intercept for each participant. The model took the form:

$$morbidity \sim \beta_1(weeks\ in\ intervention) + (1|PID) \qquad (3)$$

The within-treatment log-odds ratio reported in **Table S6** is β_1 in Equation (3) and represents how much more/less likely it would be to have a co-morbidity each week during the intervention period.

A comparison of the effects of MDCF-2 and RUSF on the prevalence of cough, rhinorrhea, fever and diarrhea was performed using a generalized mixed effects linear model with a 'logit' link function predicting a particular co-morbidity from the interaction between weeks in the intervention and treatment,

controlling for the main effects of treatment, weeks in the intervention, and a random intercept for each participant. The model took the form:

$$morbidity \sim \beta_1(treatment\colon weeks\ in\ intervention) + \beta_2(treatment) + \beta_3(weeks\ in\ intervention) + (1|PID) \quad (4)$$

The differential prevalence of co-morbidity as a function of treatment arm (MDCF-2 versus RUSF) reported in **Table S6** is β_1 in Equation (4) and represents how much more likely a given co-morbidity is to be reported each week in the MDCF-2 arm compared to the RUSF arm.

Analysis of dietary habits during supplementation

Data from a 66-question food-frequency questionnaire (FFQ) was collected at enrollment as well as one, two, four, five, nine and 12 weeks after starting the intervention. Each question prompted either a binary response (yes/no) or a count-based (integer) response. Caregivers of participants were asked to recall the foods that their children had consumed over the previous 24 hours. Food items were organized into seven groups according to WHO infant and young child feeding guidelines: (i) grains, roots and tubers; (ii) legumes and nuts; (iii) dairy products; (iv) flesh foods; (v) eggs; (vi) vitamin A rich fruits and vegetables; and (vii) other fruits and vegetables.

A comparison of the effects of MDCF-2 and RUSF on responses to FFQ items was performed using a generalized mixed-effects linear model. The underlying regression model followed either a binomial distribution for questions prompting binary answers or a Poisson distribution for questions prompting count-based answers. Each model predicted the response to a question as a function of the interaction between weeks in the study and treatment, controlling for the main effects of treatment, weeks in the intervention, as well as a random intercept for each participant. The model took the form:

$$FFQ\ response \sim \beta_1(treatment\colon weeks\ in\ intervention) + \beta_2(treatment) + \beta_3(weeks\ in\ intervention) + (1|PID) \quad (5)$$

The differential change in prevalence (for binary answers) or number of times a food group was consumed (for count-based answers) in **Table S4A** is β_1 in Equation (5) and represents how much more likely a food type was reported to be consumed in the FFQ each week in the MDCF-2 arm compared to the RUSF arm. Estimates from models that did not converge are reported as 'NA'. The overall difference in FFQ response between the two treatment arms is denoted by β_2 in Equation (5) and is reported in **Table S4B**.

Additionally, a mixed-effects linear model was created for each FFQ response to determine whether any items on the questionnaire were related to ponderal growth. Each model predicted the FFQ response as a function of WLZ, controlling for the fixed effects of treatment, weeks in the intervention, the interaction of weeks in the intervention and treatment, as well as a random intercept for each child. The model took the form:

$$WLZ \sim \beta_1(FFQ\ response) + \beta_2(treatment) + \beta_3(timepoint) + \beta_4(treatment\!:timepoint) + (1|PID) \quad (6)$$

The relationship between FFQ response and WLZ reported in **Table S4C** is β_1 in Equation (6) and represents the effect of a one-unit increase in FFQ response on WLZ. Because WLZ and FFQ responses were collected at different times, timepoints were converted to ranks (integer values between 1 through 7, reflecting the number of times WLZ was measured and FFQs were administered during the intervention) prior to building each model. Estimates from models that did not converge are reported as 'NA'.

Identification of 'WLZ-associated' proteins

Pearson correlations between changes in protein abundances and ponderal growth rates were used to nominate 'WLZ-associated' proteins. Because WLZ was measured every 15 days (a total of seven measurements throughout the course of the intervention) while plasma protein abundances were only quantified at three time points (baseline, one and three months after the start of intervention), we

developed the following strategy to maximize information used to quantify protein-anthropometry relationships. First, a linear model predicting WLZ from time in the intervention was created for each participant (n=118 who provided a full set of plasma samples), yielding 118 β-coefficients that describe the ponderal growth rate (β-WLZ) of each child from whom matched anthropometric and proteomic data were available. Next, for each participant, the change in protein abundances between the start and end of the intervention was calculated, producing 118 'Δ-abundances' for each protein. Finally, the 118 β-WLZ values were regressed against the 118 Δ-abundances for each of the 4,977 proteins that passed quality control, resulting in 4,977 Pearson correlation coefficients that captured the associations between changes in protein abundances and changes in ponderal growth. 'WLZ-associated' proteins were defined as those whose changes in abundance were significantly positively correlated with β-WLZ [FDR-adjusted p-value (q-value) less than 0.1)]. Enrichment for GO 'biological processes' was performed by rank-ordering proteins by their Pearson correlation coefficient, then performing gene set enrichment analysis (GSEA) using the *fgsea* package in R (*17*) to calculate enrichment p-values (10,000 permutations).

Differential abundance analysis of plasma proteins

Differential abundance analyses between timepoints, intervention, or the interaction between timepoint and intervention were performed using *limma* (*18*). The 'duplicateCorrelation' function, which corrects for correlations within a blocked design, was used to account for the repeated measurements taken from each participant, resulting in the equivalent of a mixed effects linear model with a random intercept for each child. Statistical significance was defined as a *limma* $q<0.1$.

Enrichment for GO 'biological processes' or the set of 70 proteins whose changes in abundances were significantly correlated with changes in WLZ was performed by rank-ordering proteins by their *limma* test-statistic (for within-treatment comparisons) or log-fold-difference (for between-treatment comparisons), then employing the *fgsea* package in R to calculate enrichment using 10,000 permutations. The term 'significant' was reserved solely for statistical tests that resulted in $p<0.05$ (e.g., enrichment for WLZ-associated proteins) or q-value<0.1 for analyses requiring correction for multiple hypotheses (e.g., enrichment for GO terms).

Analysis of gut microbiota reconfiguration

Mixed-effects linear models (R packages lme4 v1.1.23 and lmerTest v3.1.1) were used to relate the abundances of ASVs (variance-stabilizing transformed counts) in each trial participant to the same participant's anthropometric characteristics using model formulas of the form:

$$WLZ \sim \beta_1(ASV\ abundance) + \beta_2(week\ since\ baseline) + (1|PID) \quad (7)$$

ANOVA was used to determine the significance of relationships between model terms and WLZ. WLZ-associated ASVs were identified as those exhibiting false-discovery-rate adjusted p-values ≤ 0.05. Differences in abundance were calculated for each ASV in each trial participant between the beginning and end of the intervention and between the end of intervention and the one-month follow-up timepoint. These ASV responses were averaged within and compared between the (i) MDCF-2 and RUSF trial arms and (ii) participants with upper-quartile and lower-quartile β–WLZ responses. The enrichment of WLZ-associated ASVs in these comparisons was calculated using the *fgsea* package in R (10,000 permutations).

Mixed effects linear models were also used to relate the abundances of WLZ-associated ASVs to time under treatment in the current study and to time in a set of healthy, age-matched, longitudinally sampled children from a previous study in Mirpur (*7*). Reference samples (n = 309, 29 individuals) from the healthy cohort were selected based on inclusion within the upper and lower bounds of age in the current study (11-23 months) and WLZ above -1 at all timepoints. Model formulas took the following form:

$$\text{ASV abundance} \sim \beta_1(\text{month of age or month in study}) + (1|\text{PID}) \quad (8)$$

Model coefficients for age were compared between each treatment arm and the healthy cohort using z-tests, with false-discovery-rate adjusted p-values ≤ 0.05 indicating a significant result.

The durability of ASV responses was determined by comparing the beginning to end of treatment response of each taxon to the end of treatment to 1-month follow-up response in each trial participant. The effects of starting microbiota configuration and growth were assessed in the 59 children who received MDCF-2 by normalizing baseline ASV abundances with *DESeq2* and regressing them against β-WLZ.

Determining associations between food frequency questionnaire responses and ASV abundances

Mixed-effects linear models were used to determine associations between FFQ responses and the abundances of the 23 WLZ correlated ASVs. The model took the form:

$$\text{ASV abundance} \sim \beta_1(\text{FFQ response}) + \beta_2(\text{weeks since baseline}) \\ \beta_3(\text{FFQ response: weeks since baseline}) + (1|\text{PID}) \quad (9)$$

ASV abundance was parameterized by the variance-stabilizing transformed counts computed by DESeq2. β_1 represents the effect of a one-unit change in FFQ response on ASV relative abundance, while β_3 represents the rate of change of FFQ response on ASV abundance. The number of weeks since baseline was matched to the closest week in which both FFQ response and ASV data were available. β coefficients listed in **Table S16A and Table S16B** represent β_1 and β_3 in Equation (9), respectively. Q-values listed in **Table S16** represent false-discovery rate-adjusted p-values; q-values less than 0.1 are bolded and considered statistically significant. Models that did not converge are listed as “NA” in **Table S16** and were due to nearly homogenous responses to the FFQ item.

Negative binomial singular value decomposition (NB-SVD) analysis

We previously described cross-correlation singular value decomposition (CC-SVD), an analytical technique that can be used to reveal associations between disparate feature types measured in the same individual (*8*). However, because bacterial abundances measured in fecal samples from this study followed a negative binomial distribution, the statistical assumptions of CC-SVD were violated. Thus, we developed negative binomial SVD (NB-SVD), a statistical method that can be used to identify associations between disparate feature types measured from the same individual when one feature type

follows a negative binomial distribution. NB-SVD analysis begins with two abundance matrices—one for the abundances of ASVs, the other for the abundances of proteins. Each element of the ASV abundance matrix $\mathbf{A}^{MxN}$ contains $\mathbf{A}_{i,j}$—the abundance of ASV *j* in fecal sample *i*—while each element of the protein abundance matrix $\mathbf{P}^{MxP}$ contains element $\mathbf{P}_{i,k}$, which is the abundance of protein *k* in plasma sample *i*. Each row *i* represents abundances quantified in matched plasma and fecal samples taken from the same individual at the same timepoint (baseline, one month, or three months after starting intervention). All 118 participants who had available fecal and plasma samples at baseline, one month, and three months were included as rows in $\mathbf{A}^{MxN}$ and $\mathbf{P}^{MxP}$. Additionally, $\mathbf{A}^{MxN}$ was filtered to remove any ASV that was present in less than 5% of samples.

Next, a cross-association matrix between proteins and ASVs was created. For a given plasma protein *k*, negative binomial regression with Empirical Bayes shrinkage was used to predict the expected counts of each ASV from the abundance of protein *k* (*11*). This procedure was implemented using the R package *DESeq2* with the model formula '~ protein_k', a local fit for the Empirical Bayes shrinkage, and default settings for all other parameters. The output for *DESeq2* is the estimated $\log_2$(fold-change) in the expected counts for $\text{ASVs}_{1:j=N}$ for a one-unit change in the abundance of protein *k*, as well as the test-statistic (z-score) for the estimated coefficient. The reported *DESeq2* z-score for each ASV-protein relationship represents a standardized metric that quantifies the likelihood and direction of association between the abundance of bacterial ASV*j* and protein *k*. Repeating this procedure for all 4,977 proteins yields a ASV-by-protein association matrix $\mathbf{C}^{NxP}$ where each element $\mathbf{C}_{j,k}$ of the matrix is the test-statistic reported by *DESeq2* for that taxon-protein pair.

Singular value decomposition (SVD) was then performed on the association matrix $\mathbf{C}^{NxP}$ to identify distinct cross-association profiles between groups of proteins and groups of bacterial taxa. SVD is a technique that separates modes of variation into statistically uncorrelated components, called singular vectors (SVs). SVs are ordered by the amount of variation they explain about the rows and columns of $\mathbf{C}^{NxP}$; SV1 explains the most variation, SV2 explains second most, etc. SVD generates both row and column SVs, which contain the projections of the rows (ASVs) and columns (proteins) of $\mathbf{C}^{NxP}$

respectively. A projection onto an SV represents how much a given feature correlates with that SV. Because $\mathbf{C}^{NxP}$ contains the association (i.e. the negative binomial regression test-statistic calculated by *DESeq2*) between the abundances of ASVs and proteins, an SV represents a cross-association profile between these two feature types. Therefore, ASVs or proteins with the largest magnitude projections will have a cross-association profile most similar to that of the SV they most strongly project on. The most positively projecting ASVs will be strongly associated with the most positively projecting proteins and negatively associated with the most negatively projecting proteins. Similarly, the most negatively projecting ASVs will be strongly associated with the most negatively projecting proteins and negatively associated with the most positively projecting proteins. Rank-ordering features by their projections and choosing the top most positively and negatively projecting features (20 in each direction for ASVs, 50 in each direction for proteins) provided a rational way for identifying coordinated groups of bacteria and proteins whose abundances are tightly coupled.

Because SVD identifies uncorrelated components, each SV represents a *unique* cross-association profile distinct from that of other SVs. To determine the number of SVs that contain cross-association information above noise, a random-matrix approximation was employed (*19*). Briefly, $\mathbf{C}^{NxP}$ was shuffled along each column to produce a randomized association matrix without any information about the relationship between ASVs and proteins. SVD was performed on the randomized matrix, and the percent variance explained by SV1 was used as the noise threshold; any SV calculated from the SVD of $\mathbf{C}^{NxP}$ that explained less variation than SV1 of the shuffled matrix was deemed noise (**Figure S8C**). Using this method, the first 10 SVs were retained for downstream enrichment analyses.

To identify whether any of the first 10 bacterial SVs were enriched for WLZ-associated taxa, GSEA was performed on the rank-ordered ASV projections along each SV, using the list of ‘WLZ-associated’ taxa (described above) as the reference set. The same procedure was performed for protein projections to determine whether any protein SVs were enriched for WLZ-associated proteins.

DATA DEPOSITION

Bacterial V4-16S rDNA sequences in raw format (prior to post-processing and data analysis) have been deposited at the European Nucleotide Archive under study accession PRJEB38494. Code pertaining to statistical analysis is available from Gitlab. Fecal and plasma biospecimens used for the analyses described were provided to Washington University under a Materials Transfer Agreement with icddr,b.

SUPPLEMENTARY RESULTS

Effects of MDCF-2 and RUSF on mid-upper arm circumference (MUAC)

Mid-upper arm circumference (MUAC) is another measure of ponderal growth status that complements WLZ (*20,21*). MUAC improved in children in both groups during the intervention (**Table 2**). Over the 4-month period between initiation of the intervention and the end of the 1-month follow-up, children who received MDCF-2 had faster improvements in MUAC (β-MUAC) compared to those receiving RUSF (**Table 2**).

Effects of MDCF-2 and RUSF on co-morbidities

The effects of treatment on four symptoms [cough, rhinorrhea, fever and diarrhea] were quantified (*Supplementary Methods*). The prevalence of cough and runny-nose reported each week was reduced by MDCF-2 compared to RUSF supplementation (**Figure S2A,B; Table S6**). MCDF-2 administration was also associated with a greater reduction in fever (**Figure S2C**, **Table S6**). In contrast, RUSF was associated with a greater reduction in diarrhea (**Figure S2D**, **Table S6**).

Changes in dietary habits during MDCF-2 and RUSF supplementation

Over the course of the study, there was no statistically significant difference in the mean number of food groups represented in the diets of children in the MDCF-2 and RUSF arms (4.1 versus 3.88, p=0.30). More than half of the participants in both groups received an acceptable diet: at the end of the 3-month intervention, 68% (40 of 59) of children in the MDCF-2 group met the World Health Organization (WHO) requirements for minimum dietary diversity (MDD), 100% for minimum meal frequency (MMF) and 61% for minimum acceptable diet (MAD); the corresponding values in the RUSF group were 58%,

97% and 51%. No significant differences in MAD were observed between the two groups before or after the interventions.

Food frequency questionnaires were administered to mothers of study participants throughout the trial in order to monitor changes in dietary habits in addition to MDCF-2 or RUSF supplementation. Of the food frequency questionnaire items that had quantitative responses (i.e., mother asked to describe the number of times a food was consumed in the past 24 hours), six were significantly altered by treatment type over the course of the intervention; egg, sweet potato, and dairy product consumption were significantly increased, while consumption of commercially available complementary foods (e.g. cereal), legumes and the frequency of breast-feeding were significantly decreased by MDCF-2 compared to RUSF (**Figure S3A-F**, **Table S4A**). Children in the MDCF-2 trial consumed significantly more dairy products and reported a higher minimum dietary diversity compared to children in the RUSF arm (**Table S4B**). None of the items that were significantly different or that changed significantly between the two arms were associated with ponderal growth (generalized linear mixed-effects model; $p>0.05$; **Table S4C**).

Associations between food frequency questionnaire responses and ASV abundances

Linear mixed-effects models were used to determine whether changes in dietary habits, as judged by FFQ responses, were associated with the 23 WLZ-associated bacterial taxa. An affirmative answer to item FFQ101 (Are you breastfeeding your child?) was significantly positively associated with Bifidobacterium sp. ($q<0.001$) and significantly negatively associated with *Faecalibacterium prausnitzii* abundances ($q=0.04$) (**Table S16A**). The abundance of *Ruminococcus gnavus* was significantly positively associated with responses to FFQ132 (How would you describe the participant’s appetite?), which measured subjective appetite quality with higher scores indicating better appetite. Finally, abundances of *F. prausnitzii* showed a significant association with the interaction between FFQ118a (How many times did the participant consume foods made with beans, lentils, peas, corn, ground nuts, or any other legumes in the past week?) and time, indicating that consumption of legumes was associated with a faster accumulation of *F. prausnitzii* (**Table S16B**).

Relating features of the plasma proteome to members of the gut microbiota

We determined whether and how features of the plasma proteome co-vary with members of the gut microbiota, especially WLZ-associated taxa. We had previously described cross-correlation singular value decomposition (CC-SVD), a method for relating disparate features in the same individual (*8*). However, the distribution of ASV abundances measured in fecal samples from children in this study followed a negative binomial distribution, invalidating the statistical assumptions of CC-SVD. Therefore, we generalized CC-SVD to account for this distributional difference and developed negative binomial SVD (NB-SVD). We performed NB-SVD analysis by first creating an association matrix where each row represents a bacterial taxon, each column represents a plasma protein, and each element of the matrix represents the test-statistic describing how strongly plasma protein k predicts the abundance of taxon j under an Empirical Bayes negative binomial regression model - a 'correlation' equivalent for count-based data (**Figure S8A**). SVD was then performed on this association matrix to identify groups of plasma proteins that were 'correlated' with similar sets of bacterial taxa (ASVs). Each singular vector (SV) represents a unique 'association profile' between proteins and ASVs that is distinct from other SVs. ASVs with positive projections onto an SV (SV^+ taxa) show positive associations with plasma proteins with positive projections (SV^+ proteins) and negative associations with proteins with negative projections (SV^- proteins) onto that SV. Concordantly, ASVs with negative projections (SV^- taxa) show positive associations with SV^- proteins and negative associations with SV^+ proteins (**Figure S8B**).

NB-SVD revealed that of the ten SVs that carried cross-association information above noise (**Figure S8C**), SV8 was the only one that was significantly enriched for WLZ-associated taxa (GSEA p=0.002). Therefore, we focused on the plasma protein-ASV cross-association profile represented by SV8 (see **Table S14** for projections of each taxon and protein along the first 10 SVs). The association strength of the top 20 positively projecting ASVs and the top 50 most positively projecting proteins are shown in **Figure S9B** (see **Figure S10** for negatively projecting ASVs and proteins).

The top 20 taxa with positive projections on SV8 included several that were identified as having a statistically significant association with β-WLZ (e.g., *Bifidobacterium adolescentis*, *Prevotella copri*, an

Olsenella sp., and two *Blautia* sp.; **Figure S9B**). Remarkably, $SV8^+$ proteins (i.e., those that are positively associated with $SV8^+$ taxa) were significantly enriched for mediators of cartilage development and bone growth; they include SFRP4, COMP, THBS4, ROBO2, and IGF1 (discussed in *Results*), as well as collagen type VI α-3 chain (COL6A3), a key regulator of skeletal muscle development and bone density (*22,23*) (**Figure S9A,B, Table S14B, Table S15**). Additionally, the $SV8^+$ proteins were significantly enriched for members of the set of 70 WLZ-associated proteins (GSEA $p<0.001$). In contrast, $SV8^-$ proteins (i.e., those that are negatively associated with $SV8^+$ taxa) were significantly enriched for mediators of acute phase response, interleukin-6 (IL-6) activation, fatty acid oxidation, and bone resorption (**Figure S9A,B, Figure S10, Table S15**). The top 20 $SV8^-$ taxa included several *Bacteroides* sp., *Campylobacter* sp., and the *Bifidobacterium* sp. that was significantly negatively associated with β-WLZ. These bacteria were negatively associated with $SV8^+$ proteins involved in bone growth and positively associated with $SV8^-$ proteins related to inflammation, fatty acid oxidation, and bone resorption (**Figure S9A, Figure S10,** see **Table S15** for the proteins contributing most to each pathway). The results provided by NB-SVD analysis reveal that the abundances of proteins involved in bone growth and inflammation are coupled to the representation of WLZ-associated taxa, providing further evidence of potential mechanisms by which components of the gut microbiota can operate to regulate growth.

MDCF-2 responsiveness and durability of response

To further characterize mechanisms underlying the ponderal growth response to MDCF-2, we divided the cohort of children given MDCF-2 into upper and lower quartiles based on their ponderal growth rates (β-WLZ) over the course of the 3-month intervention (n=15 children/group). Those in the upper-quartile were significantly more wasted at baseline ($p=0.008$, two-sided Student's t-test), but after the first month of intervention showed complete catch-up growth to the lower-quartile responders ($p=0.82$; **Figure S6A**). By the end of the 3-month intervention, these children had significantly higher WLZ scores than those in the lower-quartile ($p<0.0001$ two-sided Student's t-test), suggesting that the differences in growth rates were not simply due to regression toward the mean (**Figure S6A**).

Comparison of the plasma proteomes of the two groups revealed that at baseline, those in the upper-quartile had higher levels of proteins involved in anti-viral immune activation including interferon α-1 (IFNA1), interferon λ-2 (IFNL2), IL-1β, IL-6, and CXCL9, and to a lesser degree, protein mediators of antimicrobial humoral immune responses (**Figure S6B, Table S10A, Table S11A**). Conversely, they had lower baseline levels of proteins belonging the set of 70 ‘WLZ-positively associated’ proteins (e.g., COMP, COL6A3, THBS3, SLITRK3, SLITRK5, and LEP) as well as other proteins assigned to the GO terms ‘bone growth’ and ‘axonogenesis’, compared to children in the lower-quartile (**Figure S6B, Table S10A, Table S11A**). These results indicate that children with a pro-inflammatory, growth mediator-depleted state at baseline exhibit the most rapid increases in WLZ during MDCF-2 treatment.

After one month of supplementation, proteins positively associated with WLZ increased, while those involved in anti-viral defense and antimicrobial immune activation decreased more in children in the upper- compared to lower-quartile (**Figure S6B, Table S10B, Table S11B)**. Interestingly, proteins associated with amino acid catabolism and fatty acid oxidation increased significantly more after one month of MDCF-2 intervention in those in the upper-quartile compared to the lower-quartile, but showed no differences at the end of treatment (**Figure S6B, Table S10B, Table S11B**). The transient metabolic shift toward amino acid utilization may reflect more successful adaptation to the additional dietary protein provided by MDCF-2 in upper-quartile individuals and is consistent with previous work on nutritional supplementation in children with severe acute malnutrition (*9*).

After three months of MDCF-2 supplementation, children in the upper-quartile of β-WLZ response had significantly elevated levels of proteins involved in bone growth and cartilage development [Matrilin-4 (MATN4), THBS3, COMP, COL6A3, and LEP] compared to those in the lower-quartile (**Figure S6B, Table S10C, Table S11C**). The inhibitory IGF binding protein IGFBP-2, and growth factor differentiation factor 15 (GDF15), which is associated with anorexia and lipolytic biomarkers in children with severe acute malnutrition (*9*), were significantly decreased in the upper quartile of MDCF-2 responders (**Table S10C**). Levels of proteins related to axonogenesis and nervous system development were also significantly increased more in the upper-quartile responders (GSEA, $q<0.1$; they include

cellular retinoic acid-binding protein 2 (CRABP2), a facilitator of the conversion of dietary carotenoids to Vitamin A (*24*), SLITRK5, NTRK2, and the axon guidance receptor UNC5B (**Table S10C, Table S11C**).

Proteins involved in antimicrobial humoral immune response exhibited significantly greater decreases after 3 months of MDCF-2 supplementation in children with upper- compared to the lower-quartile of β-WLZ responses (**Figure S6B, Table S10C, Table S11C**). The three pro-inflammatory proteins with the most statistically significant reductions were granulysin (GNLY), CXCL11 (a T-cell chemoattractant and ligand for the Th1 T-cell receptor CXCR3), and immunoglobulin A (IGHA1); others that were more decreased in the upper quartile responders (although not to a statistically significant degree after adjustment for multiple hypotheses) include the neutrophil gelatinase lipocalin-2 (LCN2, elevated in numerous inflammatory disorders; *25*), regenerating family member 1-alpha and -beta (REG1A, REG1B; elevated in the serum of children with Celiac Disease and in the feces of undernourished children at risk for stunting, respectively; *26,27*), and REG3A (highly correlated with the abundances of inflammatory proteins in the proximal small intestine of stunted children with environmental enteropathy; *2*) (**Table S10C**).

An analysis of changes in the microbiota after three months of MDCF-2 treatment revealed that children in both the upper- and lower-quartiles of β-WLZ response had significant increases in the abundances of the set of 21 positively WLZ-associated taxa (p_{upper}<0.001, p_{lower}=0.01, GSEA; **Figure S13A, Table S20**). A direct comparison between the two groups of children revealed that those in the upper-quartile of β-WLZ response exhibited larger changes in the set of 23 taxa whose abundances were significantly associated with WLZ (p=0.04, GSEA).

Figure S13B compares the microbiota configurations of children at the end of the 3-month intervention versus at the end of the 1-month follow-up period. The results show that the magnitude of change in representation of the majority of MDCF-2-responsive ASVs (16 of the 21 WLZ-associated taxa) diminished during this period, concomitant with a decline in mean WLZ (**Figure S13C**). Consistent with this finding, at the end of the 1-month follow-up period, children in the upper-quartile of β-WLZ

responses exhibited a greater drop in WLZ from the end of treatment timepoint compared to those in the lower-quartile (p=0.04, **Figure S13D**).

SUPPLEMENTARY FIGURES

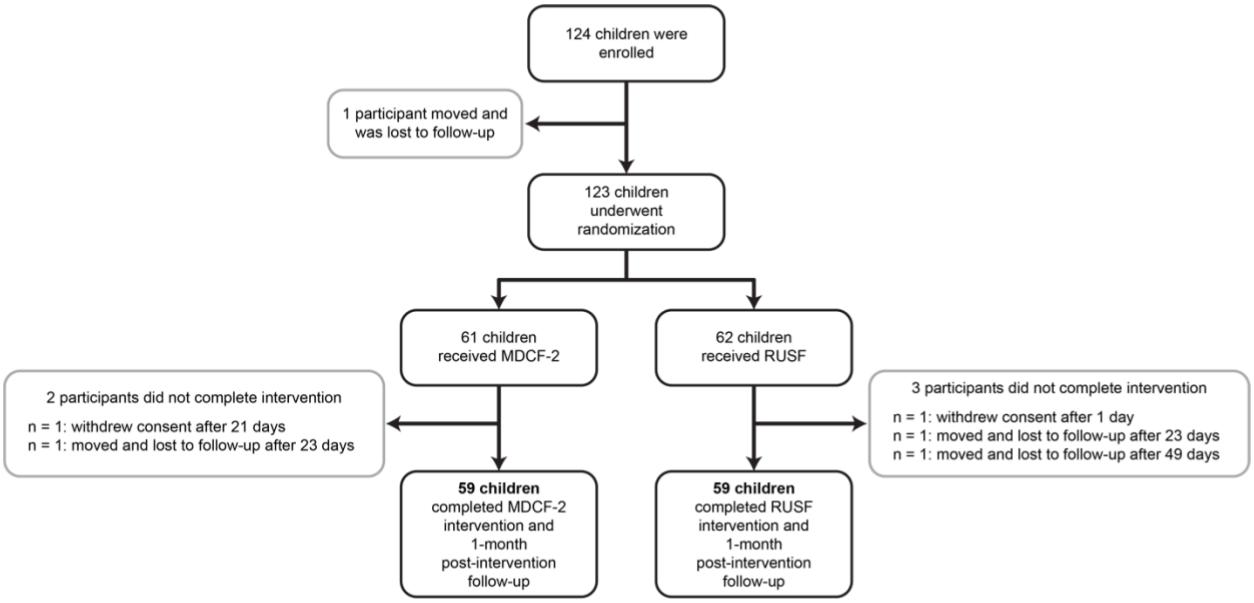

Figure S1. Study summary.

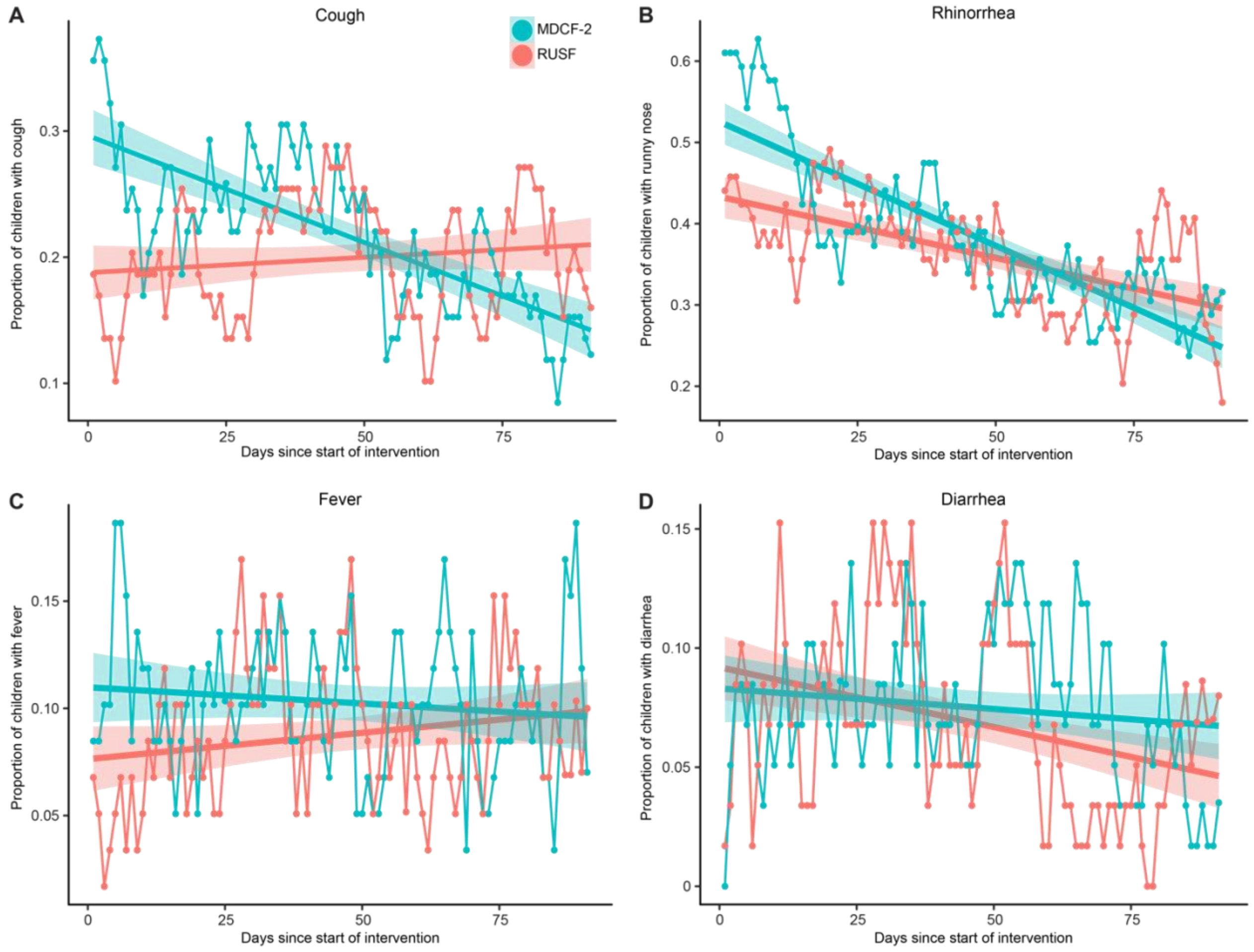

Figure S2. Effects of MDCF-2 and RUSF on illness and co-morbidities. (A-D) Change in the proportion of participants with reported cough (panel A), rhinorrhea (panel B), fever (panel C), or diarrhea (panel D) during the 3-month period of supplementation. Each dot represents the mean proportion of participants with the reported co-morbidity. Shaded regions around linear regression lines represent 95% confidence intervals.

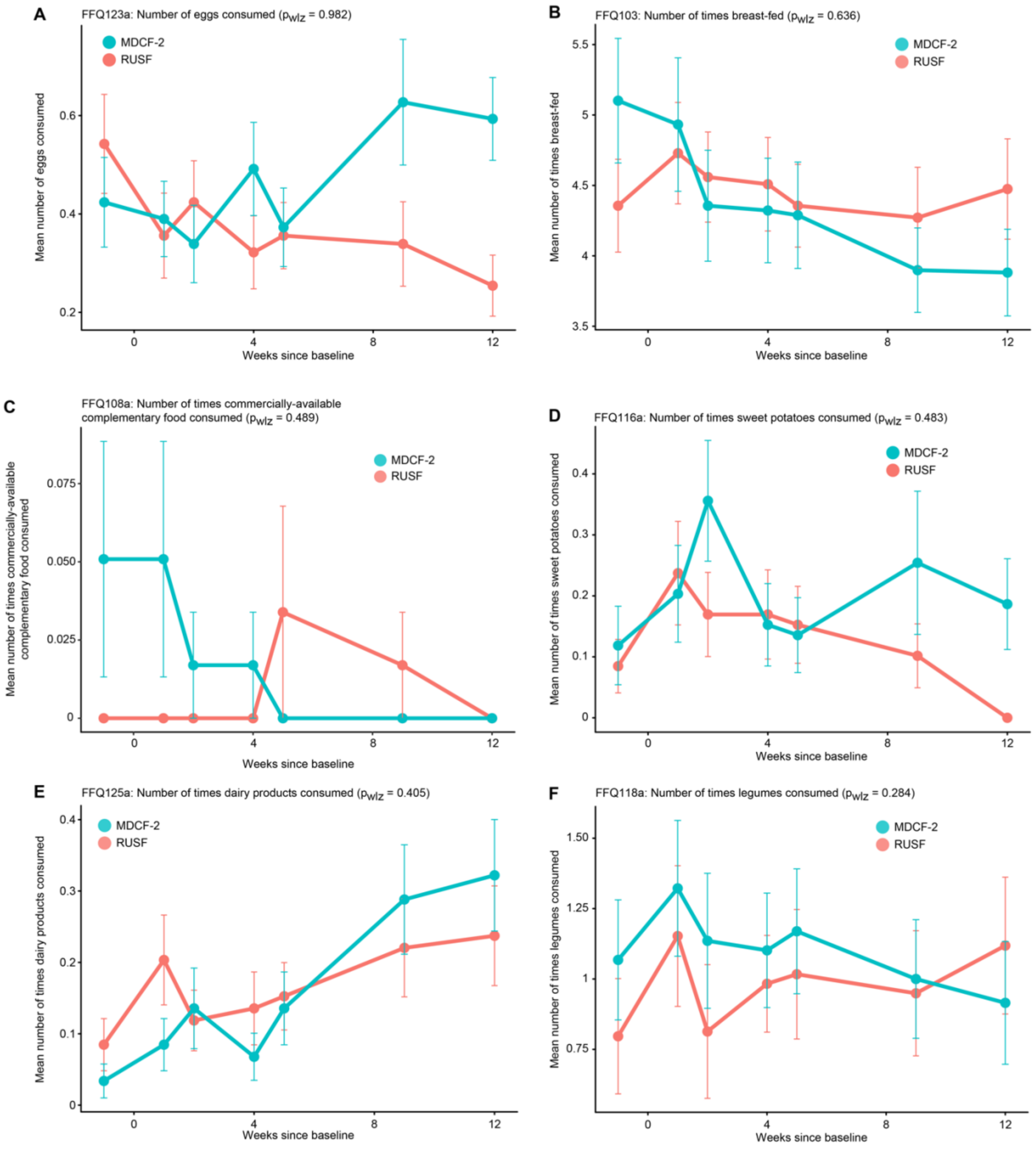

Figure S3. Food frequency questionnaire responses as a function of treatment with MDCF-2 or RUSF. (A) Number of eggs consumed. **(B)** Number of times child was breast fed. **(C)** Number of times commercially available complementary food was consumed. **(D)** Number of times sweet potatoes were consumed. **(E)** Number of times dairy products were consumed. **(F)** Number of times legumes were

consumed. Circles and error-bars denote mean and standard error. P-values in each panel header (p_{WLZ}) indicate the statistical significance of the association between FFQ response and WLZ. See **Table S4** for unadjusted P-values which indicate the interaction between treatment and weeks since starting the intervention on FFQ response.

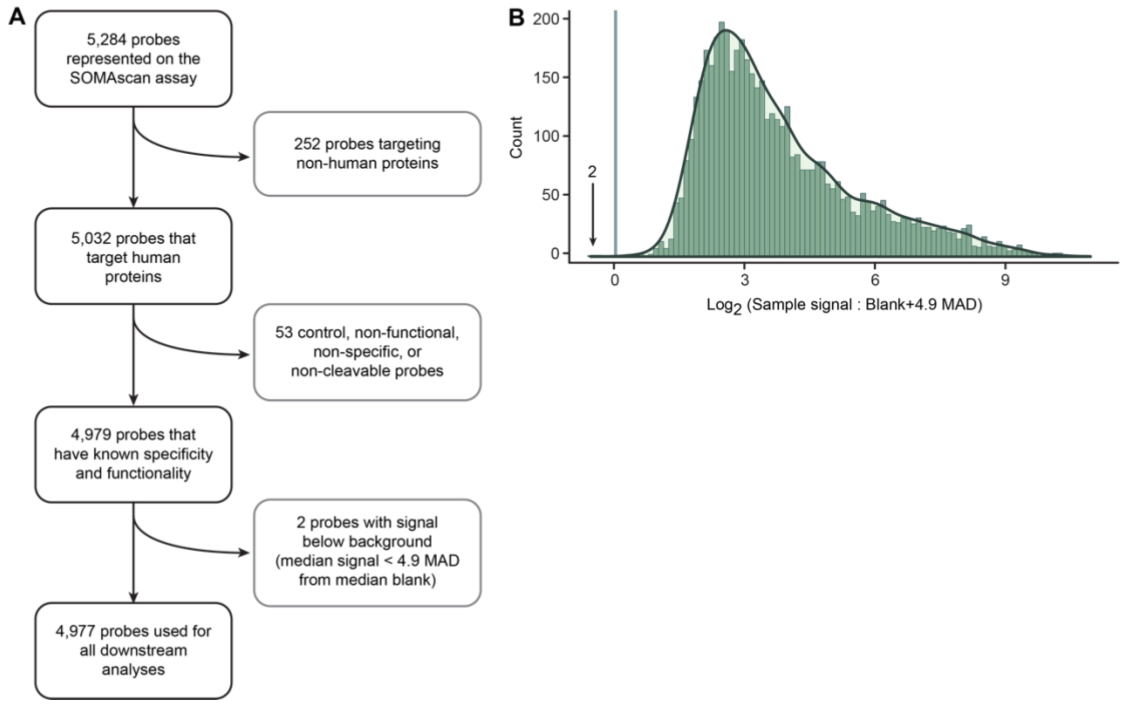

Figure S4. Quality control of proteins represented on the SomaScan platform. (A) Workflow for quality control (QC) filtering. **(B)** Distribution of signal-to-noise ratios for the SOMAmers that passed the first two QC filters. SOMAmers with median abundances across plasma samples greater than 4.9 median average deviances (MAD) from the median of blank (buffer alone) samples (indicated by the vertical line), were considered signal above noise.

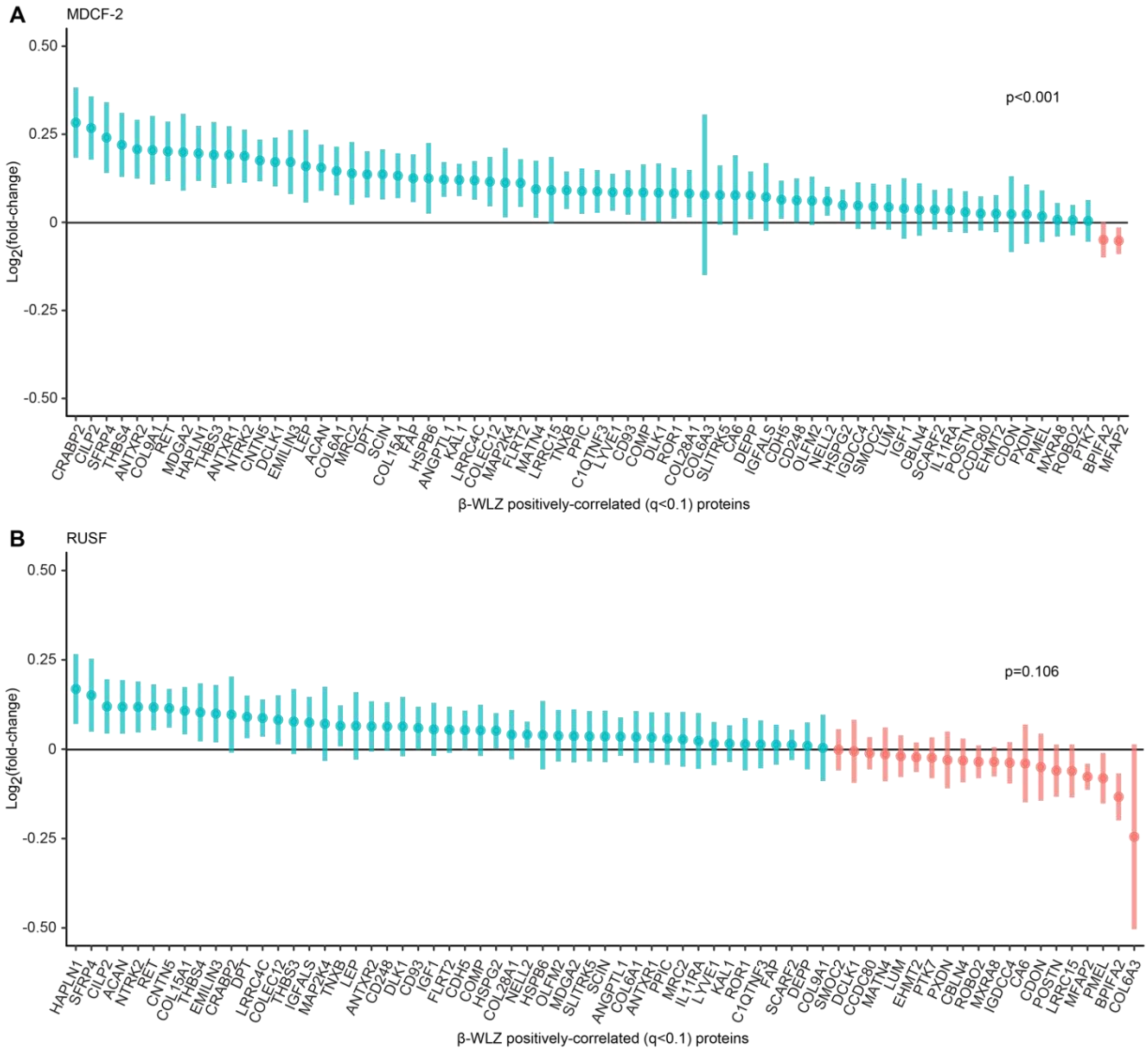

Figure S5. Effects of MDCF-2 and RUSF on WLZ-associated proteins. (A,B) Log_2(fold-changes) of 'WLZ-associated' proteins after 3-months of supplementation with MDCF-2 (panel A) or RUSF (panel B). Gene set enrichment analysis was used to calculate the enrichment of proteins whose abundances were increased after MDCF-2 or RUSF treatment for the 70 'WLZ-positively associated' proteins. Circles represent mean and error-bars represent SEM.

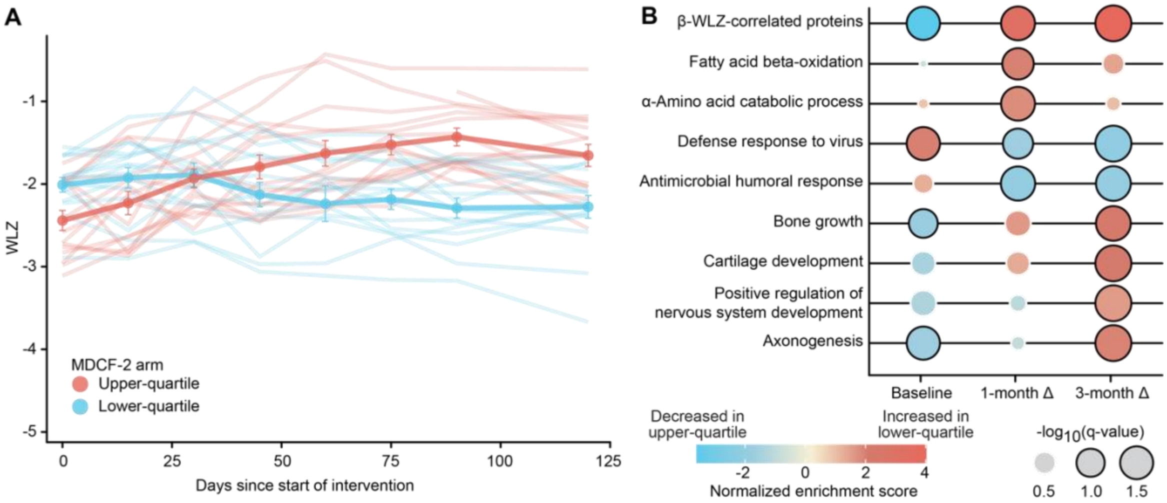

Figure S6. Analysis of MDCF-2 responsiveness. (A) Ponderal growth of participants in the upper- and lower-quartiles of β-WLZ responses. Faded lines are the WLZ trajectories of individual participants. Circles and error bars represent the mean and SEM. **(B)** Gene set enrichment analysis (GSEA) of plasma proteins that were differentially abundant at baseline, or whose changes in abundances after 1-month or 3 months of MDCF-2 supplementation were significantly different between children in the upper- and lower-quartile of β–WLZ responses. The color of each circle indicates the direction of enrichment (red, higher in upper-quartile responders; blue, lower in upper-quartile responders). The darkness of each circle represents the normalized enrichment score from GSEA. The size of each circle represents the statistical significance. Circles that are outlined in black reached the statistically significant threshold of q<0.1.

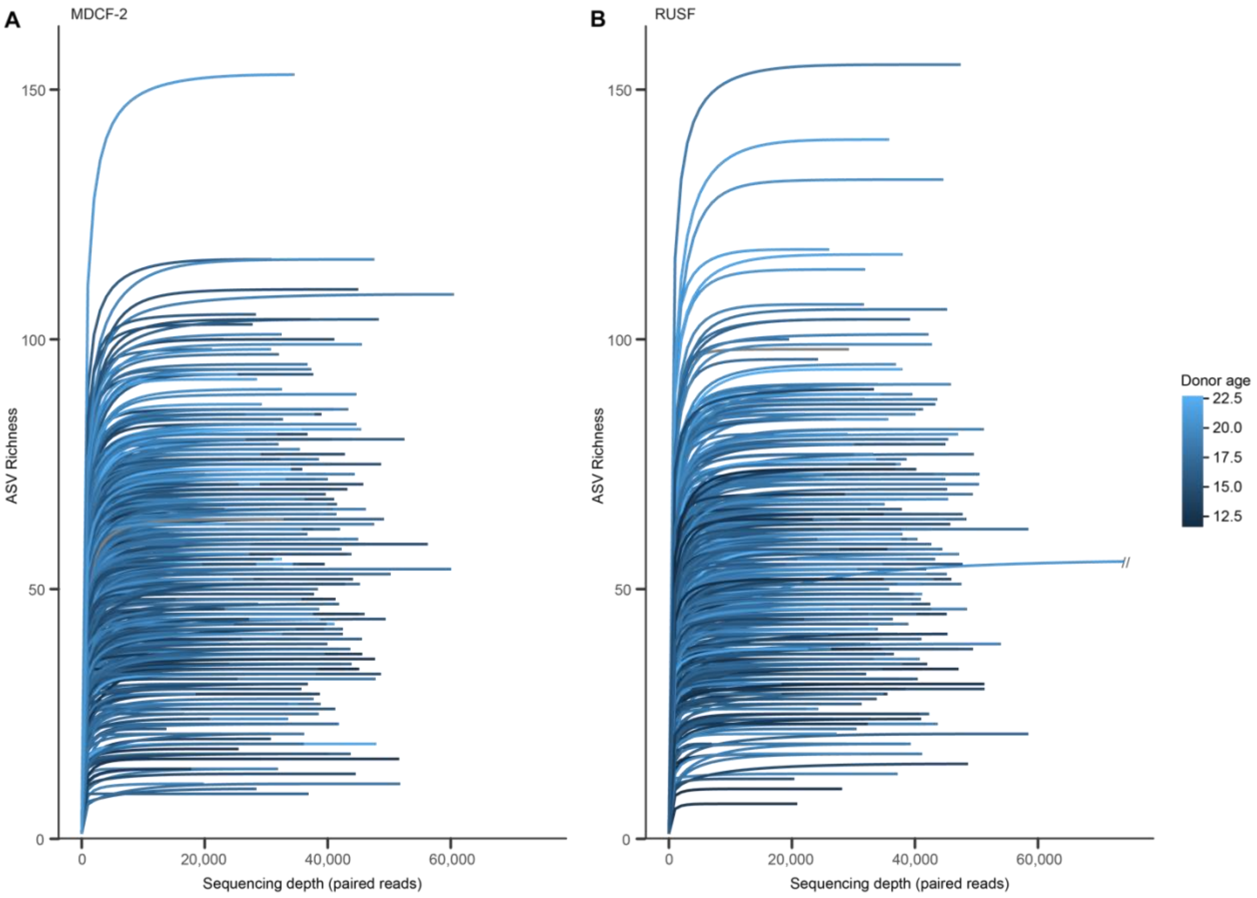

Figure S7. Analysis of V4-16S rDNA amplicon sequencing depth. Results of rarefaction analysis to determine sufficiency of amplicon sequencing depth for each sample in the (**A**) MDCF-2 and (**B**) RUSF arms of the study. Each curve quantifies the relationship of ASV richness (total number of unique ASVs) versus sequencing depth and is colored by donor age. The curve is truncated for brevity by // in panel B and extends to $4.9x10^5$ reads.

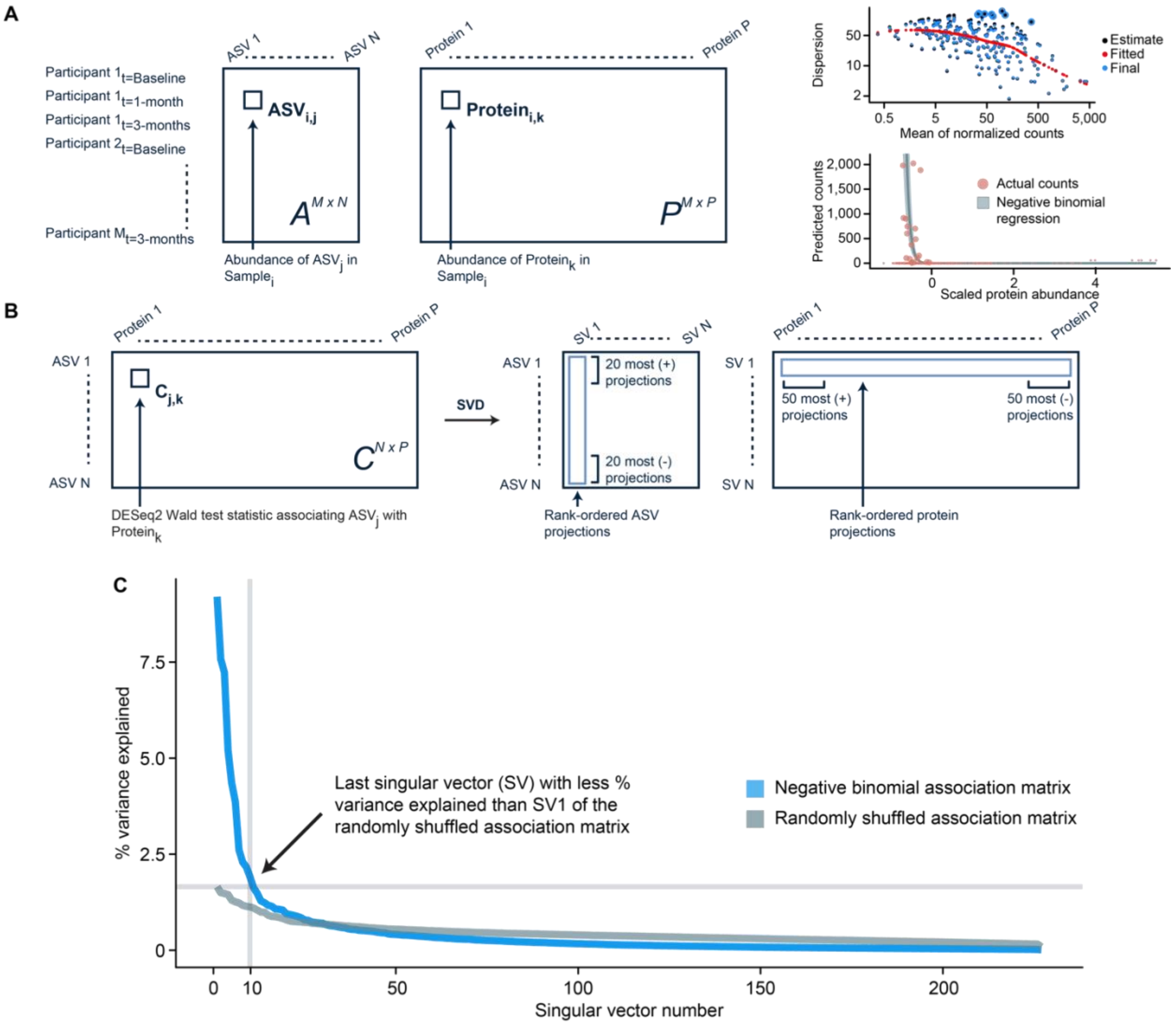

Figure S8. Method for relating features of the plasma proteome to members of the gut microbiota. (A,B) Schematic summarizing how the negative binomial cross-association matrix was created (panel A), and how negative binomial singular value decomposition (NB-SVD) was performed (panel B). Samples from each participant at baseline, one month, and three months after starting intervention were row-concatenated into bacterial ($\boldsymbol{A^{MxN}}$) or proteomic ($\boldsymbol{P^{MxP}}$) abundance matrices. For each ASV-protein pair, Empirical Bayes negative binomial regression using *DESeq2* was performed, and the resulting test statistic was stored in a cross-association matrix ($\boldsymbol{C^{NxP}}$). Singular value decomposition was performed on $\boldsymbol{C^{NxP}}$; the top most positively and negatively projecting bacterial and proteomic features along each

singular vector (SV) represent members of a unique cross-association profile that link the abundances of ASVs to the abundances of plasma proteins. **(C)** Determining the number of singular vectors with cross-association information between plasma proteins and gut bacterial taxa. SVD was performed on the cross-association matrix generated by NB-SVD analysis as well as the same cross-association matrix whose columns were randomly shuffled to remove information regarding the relationships between plasma protein and ASV abundances. The percent variance explained of each singular vector (SV) generated from decomposing the cross-association (blue curve) or shuffled (gray curve) matrix is plotted in descending order. The noise threshold was chosen to be the percent variance explained by the first SV of the shuffled matrix (horizontal line); SV10 (vertical line) from the SVD of the cross-association matrix was the last SV that explained more variance than the noise threshold.

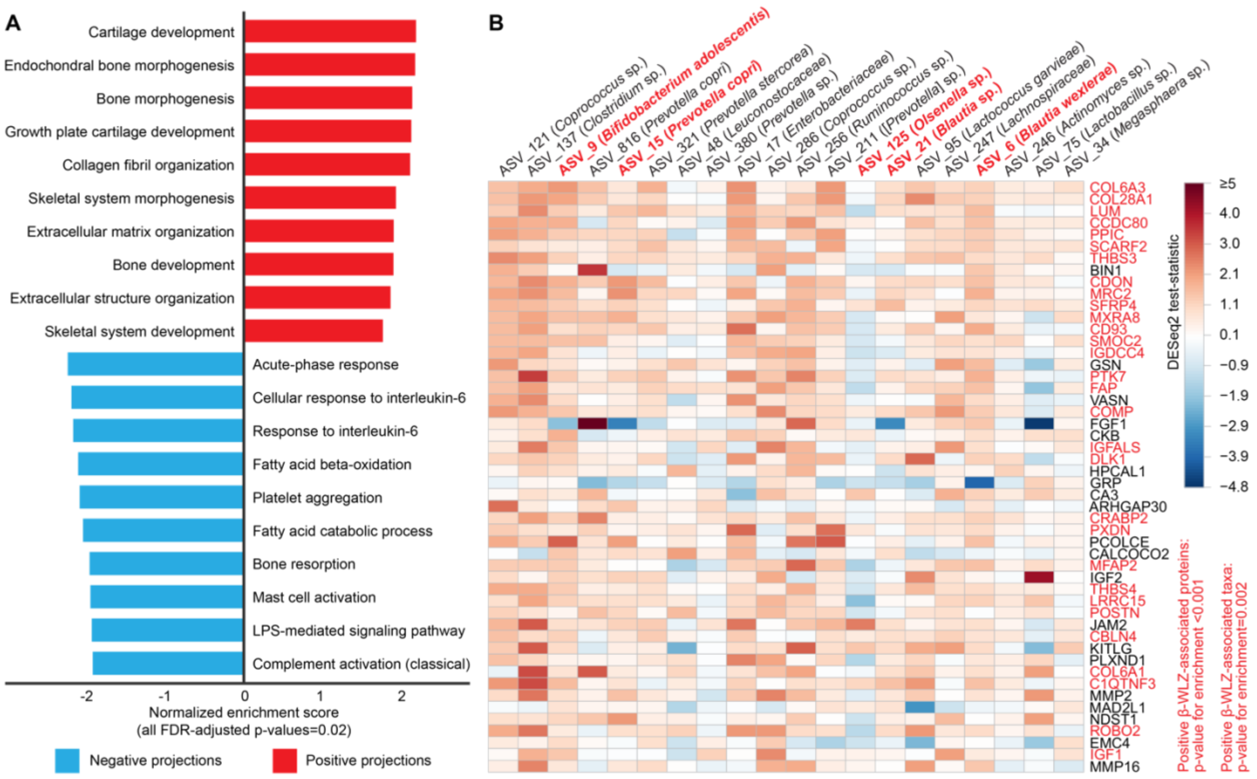

Figure S9. Results from negative binomial singular value decomposition analysis

(A) Representative GO terms from gene set enrichment analysis performed on the cross-association profile of singular vector 8 (SV8). **(B)** Heatmap of the pair-wise cross-associations (*DESeq2* test-statistics) between the top 20 and top 50 most positively projecting ASVs and proteins, respectively, along SV8. ASVs are arranged from left to right while proteins are arranged from top to bottom by decreasing projection values. The color intensity of each cell indicates the strength of association between a protein and an ASV; darker red indicates a strong positive association while darker blue indicates a strong negative association. Names of positive 'WLZ-associated' taxa and proteins are noted in red font. For example, collagen type VI α-3 chain (COL6A3), a key regulator of skeletal muscle development and bone density, and ASV_9 are strongly positively covarying and are both significantly associated with ponderal growth.

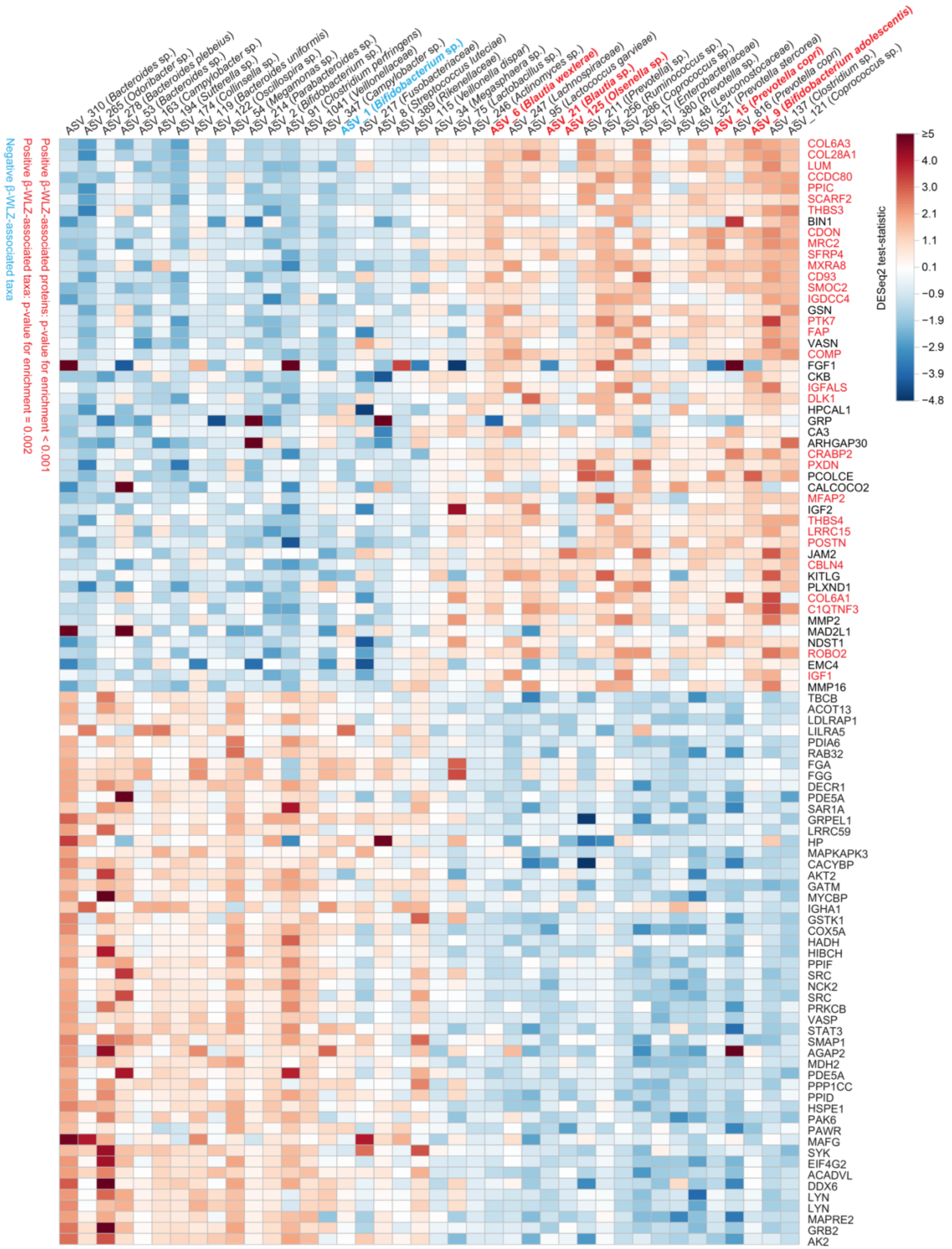
ASV_310 (Bacteroides sp.)
ASV_265 (Odoribacter sp.)
ASV_278 (Bacteroides plebeius)
ASV_53 (Bacteroides sp.)
ASV_163 (Campylobacter sp.)
ASV_194 (Sutterella sp.)
ASV_174 (Collinsella sp.)
ASV_119 (Bacteroides uniformis)
ASV_122 (Oscillospira sp.)
ASV_54 (Megamonas sp.)
ASV_214 (Parabacteroides sp.)
ASV_2 (Bifidobacterium sp.)
ASV_91 (Clostridium perfringens)
ASV_1041 (Veillonellaceae)
ASV_347 (Campylobacter sp.)
ASV_1 (Bifidobacterium sp.)
ASV_217 (Fusobacteriaceae)
ASV_8 (Streptococcus luteciae)
ASV_389 (Rikenellaceae)
ASV_115 (Veillonella dispar)
ASV_34 (Megasphaera sp.)
ASV_75 (Lactobacillus sp.)
ASV_246 (Actinomyces sp.)
ASV_6 (Blautia wexlerae)
ASV_247 (Lachnospiraceae)
ASV_95 (Lactococcus garvieae)
ASV_21 (Blautia sp.)
ASV_125 (Olsenella sp.)
ASV_211 ([Prevotella] sp.)
ASV_256 (Ruminococcus sp.)
ASV_286 (Coprococcus sp.)
ASV_17 (Enterobacteriaceae)
ASV_380 (Prevotella sp.)
ASV_48 (Leuconostocaceae)
ASV_321 (Prevotella stercorea)
ASV_15 (Prevotella copri)
ASV_816 (Prevotella copri)
ASV_9 (Bifidobacterium adolescentis)
ASV_137 (Clostridium sp.)
ASV_121 (Coprococcus sp.)
Positive β-WLZ-associated proteins: p-value for enrichment < 0.001
Positive β-WLZ-associated taxa: p-value for enrichment = 0.002
Negative β-WLZ-associated taxa
COL6A3
COL28A1
LUM
CCDC80
PPIC
SCARF2
THBS3
BIN1
CDON
MRC2
SFRP4
MXRA8
CD93
SMOC2
IGDCC4
GSN
PTK7
FAP
VASN
COMP
FGF1
CKB
IGFALS
DLK1
HPCAL1
GRP
CA3
ARHGAP30
CRABP2
PXDN
PCOLCE
CALCOCO2
MFAP2
IGF2
THBS4
LRRC15
POSTN
JAM2
CBLN4
KITLG
PLXND1
COL6A1
C1QTNF3
MMP2
MAD2L1
NDST1
ROBO2
EMC4
IGF1
MMP16
TBCB
ACOT13
LDLRAP1
LILRA5
PDIA6
RAB32
FGA
FGG
DECR1
PDE5A
SAR1A
GRPEL1
LRRC59
HP
MAPKAPK3
CACYBP
AKT2
GATM
MYCBP
IGHA1
GSTK1
COX5A
HADH
HIBCH
PPIF
SRC
NCK2
SRC
PRKCB
VASP
STAT3
SMAP1
AGAP2
MDH2
PDE5A
PPP1CC
PPID
HSPE1
PAK6
PAWR
MAFG
SYK
EIF4G2
ACADVL
DDX6
LYN
LYN
MAPRE2
GRB2
AK2
DESeq2 test-statistic
≥5
4.0
3.0
2.1
1.1
0.1
−0.9
−1.9
−2.9
−3.9
−4.8

Figure S10. Complete SV8 cross-association profile identified by NB-SVD analysis. The top 20 most positively and negatively projecting bacterial taxa and the top 50 most positively and negatively projecting plasma proteins were identified in the cross-association matrix produced by NB-SVD analysis and plotted as a heatmap. Each element represents the *DESeq2* test-statistic, a measure of association between the abundance of a bacterial taxon and plasma protein. Features are ranked by their projections along SV8. Dark red pixels indicate a strong positive association while dark blue elements indicate a strong negative association. Proteins and taxa that are positively associated with WLZ are highlighted in red. *Bifidobacterium* sp. (likely *B. longum*) is highlighted in blue and was the only WLZ-associated taxon in the top 20 negative projections along SV8.

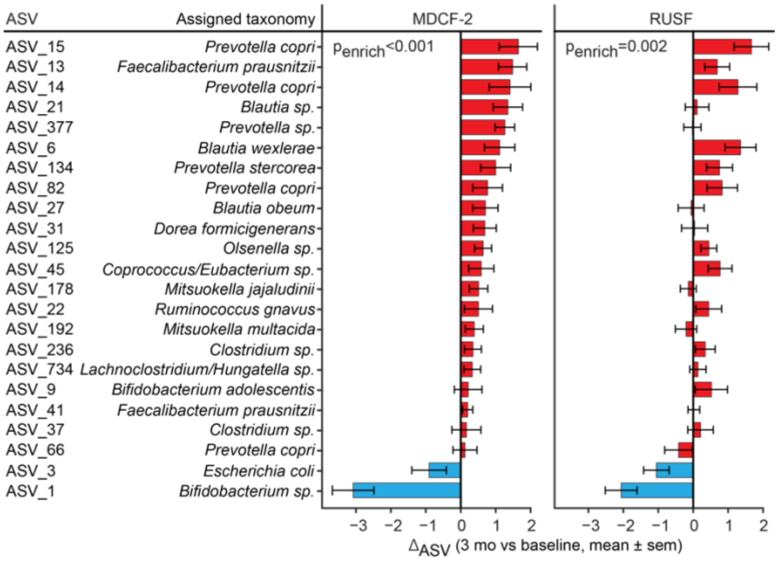

Figure S11. Effects of supplementation on WLZ-associated taxa. Changes in the abundances (variance-stabilizing transformed counts) of WLZ-associated taxa over the 3-month treatment period (‘Δ_{ASV}’) with MDCF-2 (left panel) versus RUSF (right panel). Mean values ± SEM are shown.

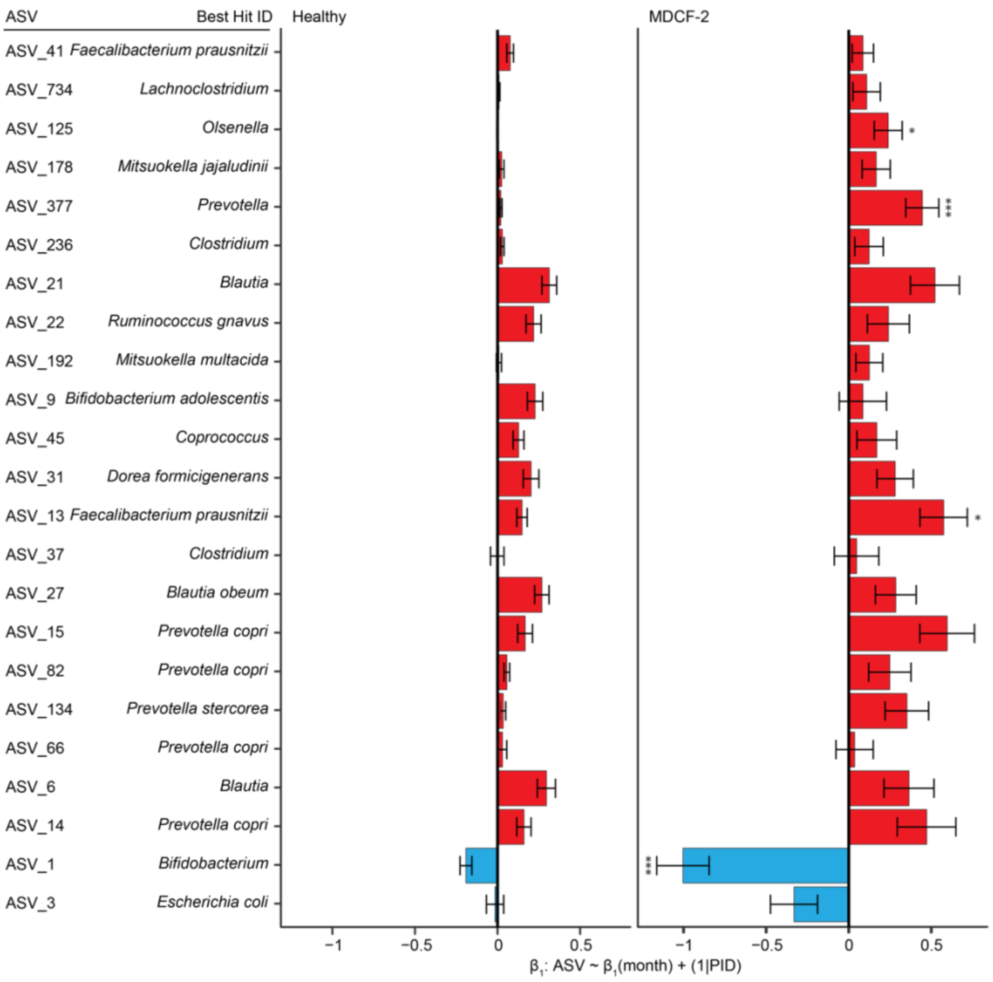

Figure S12. Comparing the rate of change in representation of WLZ-correlated ASVs in the gut microbiota of healthy Mirpur children with their representation in similarly-aged children with MAM receiving MDCF-2. Linear mixed effects models were used to determine the relationship between ASV abundance and age in children with MAM who were treated with MDCF-2 (left) or age-similar healthy Bangladeshi children (right) who were sampled monthly. Bar graphs indicate the linear model coefficients ± SEM associating ASV abundance with month for each of the 23 β-WLZ associated taxa. Statistical significance of β_1 (month) was calculated using z-tests and adjusted for false-discovery-rate. ***, $p \leq 0.001$, *, $p \leq 0.05$. Color scheme: red bars, ASVs with significant positive associations with WLZ (listed in decreasing order of the strength of association with WLZ as in **Figure 3A**); blue bars, ASVs with significant negative correlations with WLZ.

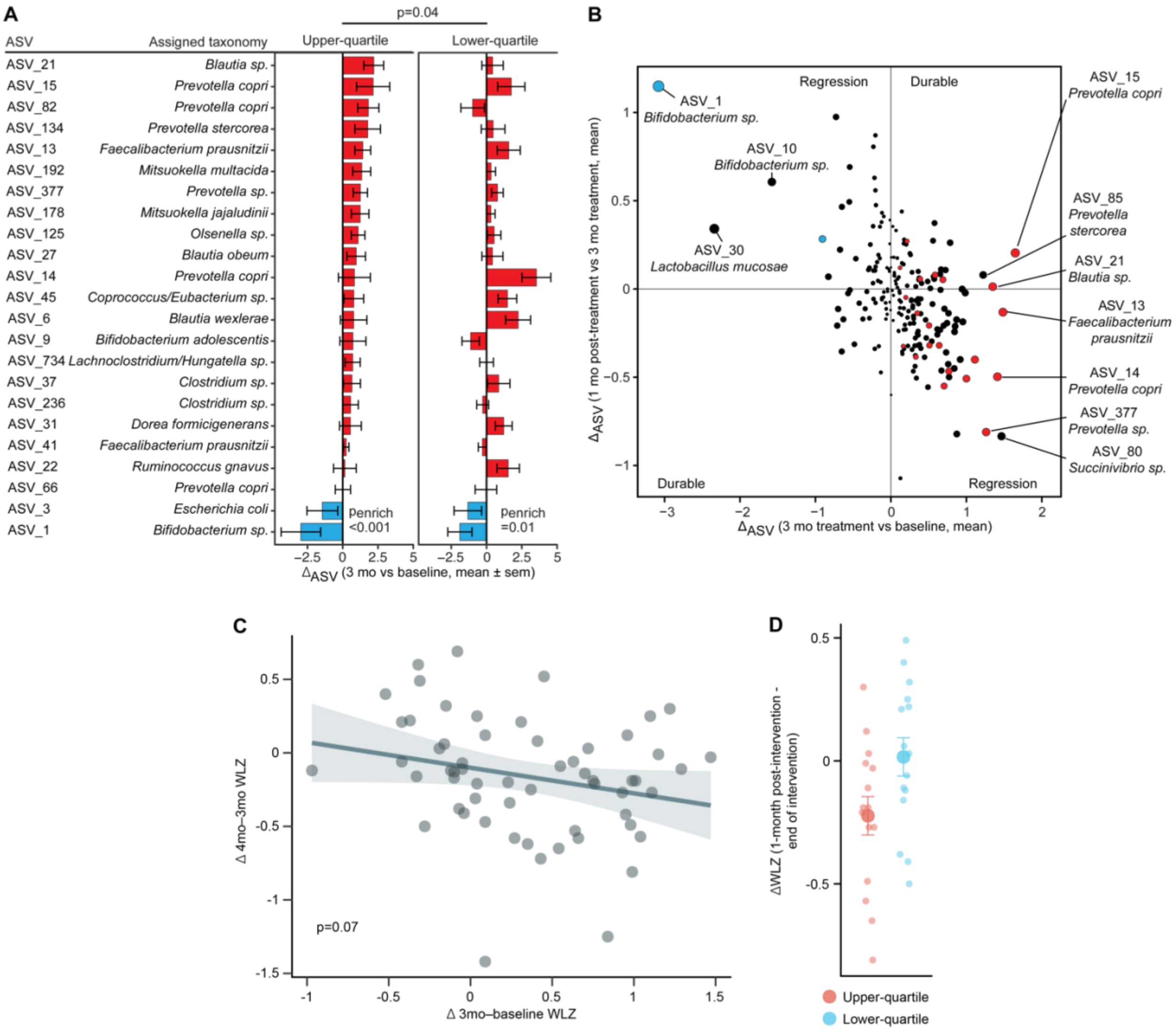

Figure S13. Determinants and predictors of MDCF-2 responsiveness. (**A**) Changes in the abundances of WLZ-associated taxa over the 3-month period of MDCF-2 treatment of children with upper-quartile (left panel) and lower-quartile (right panel) β-WLZ responses. Mean values ± SEM are shown. (**B**) Durability of microbiota response. Durability is defined by comparing (i) changes in the abundances (variance-stabilizing transformed counts) of all ASVs after 3 months of treatment with MDCF-2 (n=209 taxa that were present in at least 5% of the fecal samples surveyed) and (ii) changes in the abundances of these ASVs between the end-of-treatment and the 1-month post-treatment time point. The 10 ASVs with the greatest magnitude of positive or negative change are labeled. ASVs in the upper-left and lower-right

quadrants regressed while those in the upper-right and lower-left quadrants showed durable responses during the 1-month post-intervention follow-up. **(C)** Relationship between changes in WLZ from baseline to 3 months and between the 3- and 4-month time points. **(D)** Change in WLZ between the end of the 3-month intervention and at 1-month post-intervention timepoint. Values below zero indicate a decline in WLZ score after cessation of intervention. Small circles indicate individual participants, large circles denote mean values and error bars represent the SEM. Color legend for panels A and B: red bars/points, ASVs with significant positive associations with WLZ; blue bars/points, ASVs with significant negative associations with WLZ. Black points in panel B denote taxa that do not have significant associations with WLZ.

SUPPLEMENTARY TABLES

Table S1. Nutritional composition of MDCF-2 and RUSF.

Table S2. Ledger of enrollment characteristics and samples obtained from children in the study.

Table S3. Environmental characteristics at enrollment.

Values represent: mean ± SD; number (percentage); number (%); median [interquartile range].

Table S4. Food frequency questionnaire responses during the 3-month period of treatment. (A) Changes in food consumption between children who received MDCF-2 and RUSF. For each question, food frequency questionnaire (FFQ) response was modeled using a generalized mixed-effects linear model with a binomial distribution (for questions prompting a yes/no answer) or a Poisson distribution (for questions prompting a counts-based answer). The primary predictor was the interaction between weeks since starting the intervention and treatment, controlling for the fixed effects of time and treatment, as well as for the random intercept of each child. β is reported for the interaction between time and treatment; statistically significant β-values ($p<0.05$) are noted in bold font. Positive β indicates an increase in the proportion of participants or frequency of consumption in the MDCF-2 arm compared to the RUSF arm. (**B**) Differences in food consumption between children who received MDCF-2 and RUSF. For each question, food frequency questionnaire (FFQ) response was modeled using a generalized mixed-effects linear model with a binomial distribution (for questions prompting a yes/no answer) or a Poisson distribution (for questions prompting a counts-based answer). The primary predictor was the type of treatment, controlling for the fixed effects of time and the interaction between treatment and time, as well as for the random intercept of each child. β is reported for treatment type; statistically significant β-values ($p<0.05$) are noted in bold font. Positive β indicates an increase in the proportion of participants or frequency of consumption in the MDCF-2 arm compared to the RUSF arm. **(C)** Associations between

FFQ responses and WLZ. For each question, a mixed-effects linear model was used to predict WLZ from the FFQ response, controlling for the fixed effects of time, treatment, the interaction between time and treatment, as well as a random intercept for each child. β is reported for the main effects of FFQ response; statistically significant values ($p<0.05$) are highlighted in bold font.

Table S5. Alternative analyses of anthropometric response to MDCF-2 versus RUSF supplementation

(A) Robust linear mixed-effects modeling. β-interaction represents the differential change in anthropometric measure in the MDCF-2 arm over the RUSF arm (positive values indicate children in the MDCF-2 arm showed faster growth). Values taken from **Table 2** were computed using a standard linear mixed-effects model (LMM) and are compared against a variation of the LMM approach that is robust to outliers (Robust LMM).

(**B**) Intent-to-treat analysis.

† Values represent the mean ± SD.

‡ Linear model predicting anthropometry at the start of the intervention as a function of treatment group, controlling for baseline age, gender and any illness 7 days prior to starting the intervention. β indicates the mean difference in anthropometry between participants who were assigned to the MDCF-2 and RUSF arms at the start of the intervention. CI, confidence interval.

§ Mixed effects linear model predicting anthropometry as a function of weeks since starting nutritional supplementation, controlling for the main effects of baseline age, gender, any illness 7 days prior to starting the intervention, and a random intercept for each participant. β indicates the growth rate in unit (anthropometric measure) per week.

¤ Mixed effects linear model predicting anthropometry as a function of the interaction between treatment group and weeks since starting nutritional supplementation, controlling for the main effects of baseline age, gender, any illness 7 days prior to starting the intervention, weeks in the intervention, treatment

group, and a random intercept for each participant. β indicates the interaction between treatment and growth rate in unit/week (a positive value indicates a faster growth rate in children receiving MDCF-2).

Table S6. Effects of supplementation on co-morbidity and illness

CI: confidence interval.

† Generalized mixed effects linear model predicting prevalence of co-morbidity (binomial presence or absence) as a function of weeks since starting nutritional supplementation, controlling for a random intercept for each participant. β indicates the log-odds of reporting a co-morbidity per week; positive indicates increasing likelihood while negative indicates decreasing likelihood during intervention.

‡ Generalized mixed effects linear model predicting prevalence of co-morbidity (binomial presence or absence) as a function of the interaction between treatment group and weeks since starting nutritional supplementation, controlling for a random intercept for each participant. β indicates the increased/decreased weekly likelihood of reporting a co-morbidity in the MDCF-2 arm over the RUSF arm; positive indicates a higher likelihood while negative indicates a lower likelihood of reporting a co-morbidity in children receiving MDCF-2 compared to those receiving RUSF.

Table S7. Correlations between changes in protein abundances and ponderal growth.

Table S8. Gene set enrichment analysis of WLZ-associated proteins.

Table S9. Differential abundance analyses of the plasma proteomes of children undergoing nutritional supplementation. (A) Changes in the plasma proteome after three months of MDCF-2 supplementation. **(B)** Changes in the plasma proteome after three months of RUSF supplementation. A positive log-fold-change indicates higher protein abundance after treatment. logFC: log2(fold-change). t: *limma* test-statistic.

Table S10. Differential abundance analysis of the plasma proteomes between upper- and lower-quartile MDCF-2 β-WLZ responders. (A) Differential abundance analysis of proteins at baseline. **(B,C)** Analysis of proteins whose abundances differentially changed in children who were in the upper-versus lower-quartile of β-WLZ response after **(B)** one month or **(C)** three months of supplementation.

Table S11. Gene set enrichment analysis of differentially abundant plasma proteins between MDCF-2 upper-quartile and lower-quartile responders. (A) Gene set enrichment analysis for proteins that were differentially abundant at baseline. **(B,C)** GO terms enriched for by proteins whose changes after **(B)** one month or **(C)** three months of supplementation are different between children who were in the upper- and lower-quartile of β-WLZ response.

Table S12. qPCR-based assays of enteropathogens. Enteropathogen burden is reported in mean±SEM. Values reflect log10-transformed absolute enteropathogen abundance in (i) pg fecal genomic DNA for bacterial enteropathogens and parasites (*Clostridium difficile*, *Campylobacter jejuni/C. coli*, *Cryptosporidum*, *Entamoeba histolytica*, Enteroaggregative *E. coli*, Enteroinvasive *E. coli/Shigella*, Enteropathogenic *E. coli*, Enterotoxigenic *E. coli*, *Giardia*, and *Salmonella*), (ii) copy number for RNA viruses (Astrovirus, Norovirus GII, Rotavirus and Sapovirus), and (iii) mass of viral DNA per lysate mass for Adenovirus.

Table S13. Associations between changes in the abundances of bacterial taxa (ASVs) and rates of ponderal growth.

Table S14. Projections along the first 10 singular vectors (SV) identified from NB-SVD analysis. (A) ASV projections. **(B)** Protein projections.

Table S15. Enrichment of singular vector 8 protein projections for GO 'biological processes'.

Table S16. Associations between food frequency questionnaire responses and abundances of WLZ-correlated ASVs. (A) Association between food frequency questionnaire responses on ASV abundances. **(B)** Interaction between food frequency questionnaire responses and time on ASV abundances.

Table S17. Changes in abundances of bacterial taxa in children undergoing MDCF-2 or RUSF supplementation. Taxa in bold font are significantly correlated with WLZ.

Table S18. Linear mixed-effects modeling analysis of gut microbial configuration in healthy children or children with MAM treated with MDCF-2 or RUSF. Comparison of the rates of change in the relative abundances of the 23 WLZ-associated ASVs in healthy Bangladeshi children (longitudinally sampled each month and within the same age range as children in this study) versus children with MAM treated with MDCF-2 or RUSF. $\beta \pm$ SEM indicates the change in variance-stabilizing transformed abundance of a given taxon per month.

Table S19. Associations between baseline ASV abundances and β-WLZ in children who received MDCF-2. There were no significant associations between the abundances of 209 bacterial taxa (ASVs) that passed our filtering strategy (see *Supplementary Methods*) at the start of intervention and the rate of ponderal growth (β-WLZ) (n=118).

Table S20. Changes in the abundances of ASVs after MDCF-2 supplementation between upper-quartile versus lower-quartile β-WLZ responders. Taxa in bold-face are significantly associated with WLZ.

SUPPLEMENTARY REFERENCES

1. WHO. Technical note: supplementary foods for the management of moderate acute malnutrition in infants and children 6–59 months of age. Geneva, World Health Organization, 2012.

2. R. E. Black, R. Laxminarayan, M. Temmerman, and N. Walker, editors. 2016. Reproductive, Maternal, Newborn, and Child Health. Disease Control Priorities, third edition, volume 2. Washington, DC: World Bank. doi:10.1596/978-1-4648-0348-2. License: Creative Commons Attribution CC BY 3.0 IGO

3. L.V. Blanton, M.R. Charbonneau, T. Salih, M.J. Barratt, S. Venkatesh, O. Ilkaveya, S. Subramanian, M.J. Manary, I. Trehan, J.M. Jorgensen, Y.M. Fan, B. Henrissat, S.A. Leyn, D.A. Rodionov, A.L. Osterman, K.M. Maleta, C.B. Newgard, P. Ashorn. K.G. Dewey, J.I. Gordon. Gut bacteria that prevent growth impairments transmitted by microbiota from malnourished children. *Science* **351**, aad3311 (2016)

4. T. Ahmed, N. Choudhury, M.I. Hossain, N. Tangsuphoom, M.M. Islam, S. de Pee, G. Steiger, R. Fuli, S.A. Sarker, M. Parveen, K.P. West. Development and acceptability testing of ready-to-use supplementary food made from locally available food ingredients in Bangladesh. *BMC Pediatrics*. 2014 Dec;14(1):1-8.

5. P. Christian, S. Shaikh. A.A. Shamim, S. Mehra, L. Wu, H. Ali, R.D. Merrill, N. Choudhury, M. Parveen, R.D. Fuli. Effect of fortified complementary food supplementation on child growth in rural Bangladesh: a cluster-randomized trial. *Int. J. Epidemiol*. 2015 Dec 1;44(6):1862-76.

6. N. Lelijveld, A. Beedle, A. Farhikhtah, E.E. Elrayah, J. Bourdaire, N. Aburto. Systematic review of the treatment of moderate acute malnutrition using food products. *Matern. Child. Nutr*. 2020 Jan;16(1):e12898

7. A.S. Raman, J.L. Gehrig, S Venkatesh, H.W. Chang, M.C. Hibberd, S. Subramanian, G. Kang, P.O. Bessong, A.A.M. Lima, M.N. Kosek,W.A. Petri, D.A. Rodionov, A.A. Arzamasov, S.A. Leyn, A.L. Osterman, S. Huq, I Mostafa, M. Islam, M. Mahfuz, R. Haque, T. Ahmed, M.J. Barratt, J.I. Gordon. A sparse covarying unit that describes healthy and impaired human gut microbiota development.

Science ***365,*** eaau4735 (2019).

8. R.Y. Chen, V.L. Kung, D. Subhashish, S. Hossain, M.C. Hibberd, J. Guruge, M. Mahfuz, K.N. Begum, M.M. Rahman, S.M. Fahim, Md.A.Gazi, M.R. Haque, S.A. Sarker, R.N. Mazunder, B. Di Luccia, K. Ahsan, E. Kennedy, J. Santiago-Borges, D.A. Rodionov, S.A. Leyn, A.L. Osterman, M.J. Barratt, T. Ahmed, J.I. Gordon. Duodenal microbiota in stunted undernourished children with enteropathy. *New Engl. J. Med*. (2020).

9. J.L. Gehrig, S. Venkatesh, H.W. Chang, M.C. Hibberd, V.L. Kung, J. Cheng, R.Y. Chen, S. Subramanian, C.A. Cowardin, M.Meier, D. O'Donnell, M. Talcott, L.D. Spears, C.C. Semenkovich, B. Henrissat, R.J. Giannone, R.L. Hettich, O. Ilkayeva, M. Muehlbauer, C.B. Newgard, C. Sawyer, R.D. Head, D.A. Rodionov, A.A. Arzamasov, S.A. Leyn, A.L. Osterman, Md I. Hossain, M. Islam, N. Choudhury, S.A. Sarker, S. Huq, I. Mahnud, I. Mostafa, M. Mahfuz, M.J. Barratt, T. Ahmed, J.I. Gordon. Effects of microbiota-directed foods in gnotobiotic animals and undernourished children. *Science* **365,** eaau4732 (2019).

10. B.J. Callahan, P.J. McMurdie, M.J. Rosen, A.W. Han, A.J.A. Johnson, S.P. Holmes. DADA2: High-resolution sample inference from Illumina amplicon data. *Nat. Methods.* **13**, 581-583. (2016)

11. M.I. Love, W. Huber, S. Anders Moderated estimation of fold change and dispersion for RNA-seq data with DESeq2. *Genome Biology* **15** (2014).

12. J. Liu, J. Gratz, C. Amour, G. Kibiki, S. Becker, L. Janaki, J.J. Verweij, M. Taniuchi, S.U. Sobuz, R. Haque, D.M. Haverstick, E.R. Houpt. A laboratory-developed TaqMan Array Card for simultaneous detection of 19 enteropathogens. *J. Clin. Microbiol.* **51**, 472-80

13. J. Liu, J.A. Platts-Mills, J. Juma, F. Kabir, J. Nkeze, C. Okoi, D.J. Operario, J. Uddin, S. Ahmed, P.L. Alonso, M. Antonio, S.M. Becker, W.C. Blackwelder, R.F. Breiman, A.S.G. Faruque, B. Fields, J. Gratz, R. Haque, A. Hossain, M.J. Hossain, S. Jarju, F. Qamar, N.T. Iqbal, B. Kwambana, I. Mandomando, T.L. McMurry, C. Ochieng, J.B. Ochieng, M. Ochieng, C. Onyango, S. Panchalingam, A. Kalam, F. Aziz, S. Qureshi, T. Ramamurthy, J.H. Roberts, D. Saha, S.O. Sow, S.E. Stroup, D. Sur, B. Tamboura, M. Taniuchi, S.M. Tennant, D. Toema, Y. Wu, A. Zaidi, J.P.

Nataro, K.L. Kotloff, M.M. Levine, E.R. Houpt. Use of quantitative molecular diagnostic methods to identify causes of diarrhoea in children: a reanalysis of the GEMS case-control study. *Lancet* **388**, 1291-301 (2016).

14. J.A. Platts-Mills, J. Liu, E.T. Rogawski, F. Kabir, P. Lertsethtakarn, M. Siguas, S.S. Khan, I. Praharaj, A. Murei, R. Nshama, B. Mujaga, A. Havt, I.A. Maciel, T.L. McMurry, D.J. Operario, M. Taniuchi, J. Gratz, S.E. Stroup, J.H. Roberts, A. Kalam, F. Aziz, S. Qureshi, M.O. Islam, P. Sakpaisal, S. Silapong, P.P. Yori, R. Rajendiran, B. Benny, M. McGrath, B.J.J. McCormick, J.C. Seidman, D. Lang, M. Gottlieb, R.L. Guerrant, A.A.M. Lima, J.P. Leite, A. Samie, P.O. Bessong, N. Page, L. Bodhidatta, C. Mason, S. Shrestha, I. Kiwelu, E.R. Mduma, N.T. Iqbal, Z.A. Bhutta, T. Ahmed, R. Haque, G. Kang, M.N. Kosek, E.R. Houpt, MAL-ED Network Investigators. Use of quantitative molecular diagnostic methods to assess the aetiology, burden, and clinical characteristics of diarrhoea in children in low-resource settings: a reanalysis of the MAL-ED cohort study. *Lancet Glob Health* **6,** e1309-e1318 (2018).
15. M. Koller, W.A. Stahel. Sharpening Wald-type inference in robust regression for small samples. *Comp. Stat. Data Anal.* **55,** 2504-2515 (2011)
16. M. Koller. robustlmm: An R package for robust estimation of linear mixed-effects models. *J. Stat. Softw*. **75**, 1-24 (2016).
17. A. Sergushichev. An algorithm for fast preranked gene set enrichment analysis using cumulative statistic calculation. *bioRxiv* (2016).
18. M.E. Ritchie, B. Phipson, D. Wu, Y. Hu, C.W. Law, W. Shi, G.K. Smyth. limma powers differential expression analyses for RNA-sequencing and microarray studies. *Nucleic Acids Research* **43**, e47 (2015)
19. V. Plerou, P. Gopikrishnan, B. Rosenow, L.A.N Amaral, H.E. Stanley. A random matrix theory approach to financial cross-correlations. *Phys Rev E Stat Nonlin Soft Matter Phys.* **65** (2002)
20. A. Chiabi, C. Mbanga, E. Mah, F.N. Dongmo, S. Nguefack, F. Fru, V. Takou, V., A. Fru. Weight-for-height Z score and mid-upper arm circumference as predictors of mortality in children with severe

acute malnutrition. *J.Trop. Pediatrics* **63,** 260–266 (2017).

21. E. Grellety, M.H. Golden. Severely malnourished children with a low weight-for-height have a higher mortality than those with a low mid-upper-arm-circumference: III. Effect of case-load on malnutrition related mortality- policy implications. *Nutr. J*. **17**, 1–10 (2018).
22. M. Okada, G. Kawahara, S. Noguchi, K. Sugie, K. Murayama, I. Nonaka, Y.K. Hayashi. I. Nishimo. Primary collagen VI deficiency is the second most common congenital muscular dystrophy in Japan. *Neurology*. **69**, 1035-1042 (2007).
23. B.H. Mullin, K. Zhu, J. Xu, S.J. Brown, S. Mullin, J. Tickner, N.J. Pavlos, F. Dudbridge, J.P. Walsh, S.G. Wilson. Expression quantitative trait locus study of bone mineral density GWAS variants in human osteoclasts. *J. Bone Mineral Research*. **33**, 1044-1051 (2018).
24. J.L. Napoli, H.S. Yoo. Retinoid metabolism and functions mediated by retinoid binding-proteins. In *Methods in Enzymology* **637** (2020).
25. A.R. Moschen, T.E. Adolph, R.R. Gerner, V. Wieser, H. Tilg. Lipocalin-2: A Master Mediator of Intestinal and Metabolic Inflammation. *Trends Endocrin. Met.* **28**, 388–397 (2017).
26. R. Planas, I. Pujol-Autonell, E. Ruiz, M. Montraveta, E. Cabre, A. Lucas-Martin, R. Pujol-Borrell, E. Martinez-Caceres, M. Vives-Pi. Regenerating gene Iα is a biomarker for diagnosis and monitoring of celiac disease: A preliminary study. *Trans. Res.***158,** 140–145 (2011).
27. K.M. Peterson, J. Buss, R. Easley, Z. Yang, P.S. Korpe, F. Niu, J.Z. Ma, M.P. Olortegui, R. Haque, M.N. Kosek, W.A. Petri. REG1B as a predictor of childhood stunting in Bangladesh and Peru. *Am. J. Clin. Nutr*. **97**, 1129–1133 (2013).
